# Comparison of efficiency and specificity of CRISPR-associated (Cas) nucleases in plants: An expanded toolkit for precision genome engineering

**DOI:** 10.1371/journal.pone.0211598

**Published:** 2019-02-27

**Authors:** Oleg Raitskin, Christian Schudoma, Anthony West, Nicola J. Patron

**Affiliations:** Earlham Institute, Norwich Research Park, Norwich, Norfolk, United Kingdom; ICAR-National Research Centre on Plant Biotechnology, INDIA

## Abstract

Molecular tools adapted from bacterial CRISPR (Clustered Regulatory Interspaced Short Palindromic Repeats) systems for adaptive immunity have become widely used for plant genome engineering, both to investigate gene functions and to engineer desirable traits. A number of different Cas (CRISPR-associated) nucleases are now used but, as most studies performed to date have engineered different targets using a variety of plant species and molecular tools, it has been difficult to draw conclusions about the comparative performance of different nucleases. Due to the time and effort required to regenerate engineered plants, efficiency is critical. In addition, there have been several reports of mutations at sequences with less than perfect identity to the target. While in some plant species it is possible to remove these so-called 'off-targets' by backcrossing to a parental line, the specificity of genome engineering tools is important when targeting specific members of closely-related gene families, especially when recent paralogues are co-located in the genome and unlikely to segregate. Specificity is also important for species that take years to reach sexual maturity or that are clonally propagated. Here, we directly compare the efficiency and specificity of Cas nucleases from different bacterial species together with engineered variants of Cas9. We find that the nucleotide content of the target correlates with efficiency and that Cas9 from *Staphylococcus aureus* (SaCas9) is comparatively most efficient at inducing mutations. We also demonstrate that 'high-fidelity' variants of Cas9 can reduce off-target mutations in plants. We present these molecular tools as standardised DNA parts to facilitate their re-use.

## Introduction

Components of bacterial CRISPR (Clustered Regulatory Interspaced Short Palindromic Repeats) systems for adaptive immunity have been repurposed for engineering the genomes of eukaryotic organisms [1–3]. These molecular tools have been rapidly and successfully applied in many organisms, including plants (for recent reviews see [4–6], primarily due to the ease at which they can be programmed to recognise new genomic targets. The majority of plant genome engineering studies have utilised Cas9 (CRISPR Associated Protein 9) from *Streptococcus pyogenes* (SpCas9), a monomeric nuclease found in the Type II CRISPR system of that species. The Cas9 protein can be directed to selected genomic targets by an engineered RNA moiety known as the single guide RNA (sgRNA) [1,2]. One or more sgRNAs can be programmed to recognise new genetic targets by recoding the ~19 base pairs at the 5' end of the RNA molecule, known as the spacer [7]. The sgRNA forms a ribonuclease complex with Cas9 enabling it to scan DNA, pausing when it encounters a cognate sequence known as the protospacer adjacent motif (PAM) [8]. On recognition of a cognate PAM, the ribonuclease complex probes the adjacent sequence to determine identity to the spacer region. If complementary, the spacer region of the sgRNA forms a Watson-Crick base-pair with the target DNA forcing the Cas9 protein to undergo a conformational change that enables the nuclease domains to cleave each of the DNA strands [8]. The most common application in plants has been targeted mutagenesis achieved following transgenic expression of sgRNA-guided Cas9 to introduce double strands breaks (DSBs) at selected genomic loci. These induced breaks are predominantly repaired by the endogenous mechanism of non-homologous end-joining (NHEJ), which sometimes introduces errors [9,10]. Since a perfect repair will continue to be recognised by the Cas9/sgRNA complex, and therefore cut again, constitutive expression eventually results in mutations, typically small insertions or deletions at the target. In many transgenic events, these mutations occur sufficiently early in the development and regeneration of the transgenic plant that all cells of the plant contain the same mutant genotype. A smaller number of studies have successfully leveraged the induction of targeted DSBs to increase the efficiency of targeted integration via the mechanism of homology directed repair (HDR), enabling transgenes to be inserted at a precise locus or for genomic DNA sequences to be specifically recoded [11–16].

Additional Cas proteins from other bacterial species have been adapted for genome engineering in eukaryotes and have also been applied to plants. These include Cas9 from the *Staphylococcus aureus* Type II CRISPR system (SaCas9) [17–19] and Cas12a (previously Cpf1) from the Type V CRISPR systems found in *Francisella novicida*, *Acidaminococcus sp*. *and Lachnospiraceae bacterium* [3,14,20,21] (FnCas12a and LbCas12a). Cas proteins from different species generally show preferences for different PAMs and, therefore, these additional Cas proteins have increased the number of genomic sites that can be targeted for engineering. While SpCas9 most efficiently cleaves DNA adjacent to NGG PAMs, SaCas9 is reported to show preference for NNGRRT [22–24]. Cas12a-RNA complexes efficiently cleave target DNA preceded by a short T-rich PAM, with data suggesting a preference for TTTV [3,25]. In addition, engineered versions of Cas9 and Cas12a with mutations in their PAM recognition domains have further expanded the repertoire of target sites to include sequences adjacent to NGAG and NGCG PAMS (with variants of SpCas [26] and NNNRRT PAMs (with variants of SaCas9 [22]).

Cas-mediated genome engineering in plants has often utilised established transformation methods (e.g. *Agrobacterium*-mediated) to deliver DNA molecules encoding expression cassettes for the Cas9 protein, one or more guide RNAs and, typically, a plant selectable marker cassette, to the plant cell aiming for integration of all components at a single genetic locus. To achieve this, several systems have been developed for the assembly of multigene constructs, many of which employ Type IIS restriction cloning, also known as Golden Gate assembly [11,27–31]. Following the recovery of transgenic plants, most studies report the 'efficiency' of targeted mutagenesis as the percentage of transgenic plants in which mutations are found at the intended target. Most published studies have utilised different genetic targets, construct designs and regulatory elements making it difficult to draw conclusions about the comparative efficiencies and specificities of each nuclease. A few recent studies have attempted to compare Cas9 and Cas12a nucleases in similar experimental conditions (e.g.[32]. However, they have necessarily used different targets with each type of nuclease, which are known to exert significant influence on efficiency [33].

Another area of interest has been the specificity of Cas nucleases. With careful bioinformatic analysis it is often possible to identify target sites in the genomic region of interest that are unique within the genome, thereby reducing the likelihood of mutations being induced at alternative loci. However, a number of different studies, including several in plants, have reported so-called "off-target" mutations at sites in the genome with less than perfect identity to the spacer [34–36]. In some cases, small numbers of off-target mutations are of no concern, especially as mutations are known to occur naturally during the process of tissue culture and regeneration [37,38]. Further, many species can be back-crossed to the parent to remove unwanted mutations. However, when the aim is to induce mutations in specific members of a gene family, specificity is desirable, especially for recent paralogues that may be in close proximity and therefore impossible to segregate. In addition, the introduction of additional unwanted mutations in lineages that are not typically sexually propagated, such as cultivars of *Solanum tuberosum* (potato), is particularly undesirable.

To increase the specificity of Cas9 proteins for their target, researchers have engineered the nuclease and PAM-recognition domains [39–41]. The resulting proteins were initially tested in mammalian cell cultures and reported to maintain similar levels of efficiency to the wild-type protein at sequences with an exact match to the spacer, but limited activity at sequences with less than perfect identity to the spacer. Here we report the outcomes of experiments that compare the efficiency and specificity of multiple wild-type (SpCas9, SaCas9, FnCas12a and LbCas12a) and engineered variants of Cas nucleases (eCas9 1.0, eCas9 1.1, eSaCas9 [39] and xCas9 3.7 [40]). All constructs used exactly the same regulatory elements and are assembled into identical vectors for delivery. Importantly, the efficiency and specificity of each nuclease are initially compared at the same target and, by quantifying the frequency of mutagenesis induced during transient expression, we avoid the influence of transgene insertion location on transgene expression. We also present an analysis of all potential targets as well as their off-targets in the coding exons of Arabidopsis and test our tools at a larger number of targets to identify factors that influence efficiency.

## Methods

### Analysis of targets in Arabidopsis coding sequences

Coding sequences were extracted from the Arabidopsis thaliana TAIR10 annotated whole chromosome datasets (ftp://ftp.arabidopsis.org/home/tair/Sequences/whole_chromosomes/ and https://www.arabidopsis.org/download_files/Genes/TAIR10_genome_release/TAIR10_gff3/TAIR10_GFF3_genes.gff). Jellyfish (v.2.2.6) [42] was combined with a script (available at https://github.com/EI-CoreBioinformatics/CRISPRanto) to extract all possible legitimate candidate target sequences for each Cas nuclease [N_20_NGG (SpCas9), N_21_-NNGGGT (SaCas9), N_20_-NGAG (SpCas9-VQR), N_20_-NGCG (SpCas9-VRER), TTTV-N_23_ (Lb/AsCas12a)]. Potential off-targets (target sequences that are either identical to another target or differ by, at most, one base pair in the spacer region) were detected by mapping all identified targets against the TAIR10 genomic sequences using bbmap (v.38.06) with the following parameters (k = 8, minid = 0.75, ambig = all, mappedonly = t, secondary = t, sssr = 0.75, ssao = t, saa = f, mdtag = t, nhtag = t, xmtag = t, amtag = t, nmtag = t, xstag = t, indelfilter = 0, subfilter = 4, editfilter = 4). Alignments were filtered by Hamming distance allowing at most one mismatch in the spacer (plus mismatches at the ambiguous positions of the respective PAM sequences). Any potential off-targets located in coding sequences were identified with Bedtools intersect (v. 2.26.0; [43] requiring an overlap equal to the length of the target including the PAM. All identified targets are provided S1 Table. All scripts and a snakemake pipeline [44] containing the whole workflow are available at https://github.com/EI-CoreBioinformatics/CRISPRanto.

### Selection of targets and off-targets for assessment of targeted mutagenesis

To remove any potential variability that might be associated with gene expression, we selected targets/off-target pairs located in genes expressed in leaves. To do this, we compared our candidate target/off-target pairs to gene expression data from the Expression Atlas (https://www.ebi.ac.uk/gxa/home, experiment E-GEOD-38612), retaining those targets within genes expressed in leaves. We then selected candidate target/off-target pairs that differed within the first 3 bp distal to the PAM (S2 Table). Finally, we either selected target/off-target pairs in which the expected cleavage site overlapped with the recognition site for a Type II restriction endonuclease, or searched 1000 bp of sequence each side of the target/off-target pairs to identify the presence of an additional target, identical in both sequences, that would enable the creation of a deletion when a second sgRNA recognising this target was delivered.

### Assembly of constructs

Constructs were assembled using the Plant Modular Cloning (MoClo) plasmid toolkit [45], a gift from Sylvestre Marrillonet (Addgene Kit # 1000000044). New Level 0 parts were made according to the standards described in [46]. All Level 0 parts encoding Cas9 proteins contained a nuclear localisation (NLS) KKRKVKKRKVKKRV signal and were assembled together with a C-terminal yellow fluorescent protein (YFP). Cas12a proteins were fused to the same C-terminal NLS tag, followed by a 3xHA tag (a YFP tag was not used as it has not been determined if this reduces nuclease activity). Level 0 parts were assembled into transcriptional units in Level 1 acceptor plasmids in a one-step digestion-ligation reaction, except for sgRNAs, in which a U6 promoter L0 part was assembled into Level 1 acceptor plasmid together with a PCR amplicon of the complete sgRNA (the spacer is fused to the RNA scaffold as a 5' extension of the forward primer), as described in [35]. Subsequently, Level 1 transcriptional units were assembled into the Level 2 acceptor plasmid pICSL4723 (Addgene #86172). The digestion-ligation reactions were set up either manually or at nanoscale using laboratory automation. For manual assembly, 15 fmol of each DNA part was combined with 7.5 fmol of the acceptor plasmid in a final volume of 5 μL dH_2_0. This was combined with 5 μL of reaction mix (3 μL of dH_2_0, 1 μL of T4 DNA ligase buffer 10x (NEB, Ipswich, MA, USA), 0.5 μL of 1 mg/mL purified bovine serum albumin (1:20 dilution in dH_2_0 of BSA, Molecular Biology Grade 20 mg/mL, NEB), 0.25 μL of T4 DNA ligase at 400 U/μL (NEB) and 0.25 μL of *BsaI* or *BpiI* restriction enzyme at 10 U/μL (ThermoFisher, Waltham, MA, USA)) and incubated in a thermocycler for 26 cycles of 37°C for three minutes followed by 16°C for four minutes and a final incubation at 37°C for 5 minutes followed by 80°C for five minutes. A 2 μL aliquot of each reaction was transformed into 20 μL electrocompetent JM109 or DH5alpha *Escherichia coli* cells and plated on selective LB-agar plates. Automated reactions were scaled down to a final reaction volume of 1 μL using the Echo 550 liquid handler (Labcyte Ltd. San Jose, SA, USA), transformed into 2 μL XL10-Gold Ultracompetent *E*. *coli* (Agilent Technologies, Santa Clara, CA, USA) and plated onto eight-well selective LB-agar plates on a Hamilton STARplus platform. The sequences of assembled plasmids were verified by complete sequencing using 150 base pair paired-end reads on an Illumina MiSeq platform. Libraries were prepared using the Nextera XT DNA Library Prep Kit (Illumina, San Diego, CA, USA) with a modified 2 μL total volume protocol using a one in 25 dilution of components. A complete list of all 132 plasmids, comprising Level 0 DNA parts, Level 1 transcriptional units and Level 2 constructs, used in this study is given in S2 Table. Samples, together with complete annotated sequence files for the 128 new plasmids generated for this study have been deposited at the AddGene plasmid repository.

### Transient expression in protoplasts

For each experiment, a sufficient number of protoplasts were prepared to enable the delivery of four replicates of all constructs to be compared. Protoplasts were prepared from the leaf tissues of *Nicotiana benthamiana* or *A*. *thaliana* as previously described [47]. Protoplasts were quantified and divided into aliquots of 200 **μ**L in transfection buffer (0.4M mannitol, 15mM MgCl_2_, 4 mM MES, pH 5.6), each containing approximately 1 x 10^4^/mL intact protoplasts, such that four separate aliquots of protoplasts from the same preparation were available for each of the plasmids to be compared. Plasmid DNA for delivery to protoplasts was prepared using the Plasmid Plus Midi kit (Qiagen, Hilden, Germany) with a modified protocol incorporating three additional wash steps prior to elution from the column. Freshly made PEG (2 g of PEG (Mn 4000 (Sigma, 81240)) in 2 mL of 500 mM mannitol and 0.5 mL of 1M CaCl_2_) was mixed with10 μg of purified DNA and added to each aliquot of protoplasts. Subsequently, protoplasts were washed and resuspended in 300 μL of washing buffer (154 mM NaCl, 125 mM, CaCl_2_, 5 mM KCl, 2 mM MES; pH5.6) and incubated for 24 hours at 24°C in an illuminated incubator with light intensity of approximately 70 **μ**mol/m^2^/s. Transformation efficiency was estimated by quantification of protoplasts in which YFP fluorescence was visible in the nuclei using an inverted fluorescence microscope (Zeiss Axio Observer Z1 or ThermoFisher Evos).

### Detection and quantitation of targeted mutagenesis

DNA was extracted from the protoplasts using a cetyltrimethylammonium bromide (CTAB) extraction protocol. Pellets of protoplasts were resuspended in 100 **μ**L of extraction buffer (0.2M Tris-HCl, pH7.5; 0.05M EDTA; 2M NaCl; 2% CTAB, pH7.4) and incubated at 65°C for 1 hour prior to addition of 45 **μ**L chloroform. Following centrifugation, the upper aqueous phase was precipitated with an equal volume of isopropanol. DNA pellets were washed with 70% w/v ethanol, dried and resuspended in sterile distilled water with 5 μg/μL RNAse A (Thermo Fisher). Each target was amplified using a pair of primers, specific to the locus of interest (S3 Table). PCR reactions were performed using 70 ng DNA and Q5 High-Fidelity DNA Polymerase (NEB) according to the instructions provided by the manufacturer. Mutations at the targets were identified by either Illumina or Sanger sequencing. For preparation for Illumina sequencing, amplicons were purified using Agencourt AMPure XP (Beckman Coulter) and indexed using the Nextera XT Library Preparation kit (Illumina) according to the manufacturer's instructions. Reactions were analysed by microfluidic gel fractionation (LabChip GXII, Perkin Elmer) and pooled. Primers were removed by fractionation (BluePippin, SAGE Science). The concentration and quality of DNA was analysed by QUBIT (ThermoFisher) and qPCR (Kapa Library Quantification Kit, Illumina). phiX Sequencing Control V3 (Illumina) was added to final concentration of 1.75 pM. Sequencing was performed on an Illumina MiSeq using the MiSeq Reagent Kit v2 Micro. Adapter sequences were removed and quality trimmed using bbduk (v.37.24) (https://jgi.doe.gov/data-and-tools/bbtools/bb-tools-user-guide/bbduk-guide/) (parameters ktrim = r k = 21 mink = 11 hdist = 2 qtrim = lr trimq = 3 maq = 10 ftr = 250). All sequences have been deposited in the EMBL Nucleotide Sequence Database (ENA) accession number = PRJEB3044. Quantification of mutations at the target was performed using CRISPRESSO (http://crispresso.rocks/) [48] and automated with a custom script (available at https://github.com/EI-CoreBioinformatics/CRISPRanto). The quantity of mutations in each sample was then normalised to the quantified transfection efficiency.

For analysis by Sanger sequencing, amplicons were purified (Qiaquick PCR purification, Qiagen) and incubated with a restriction enzyme for which the recognition sequence overlapped the expected cleavage site prior to reamplification. This reduced the amount of wild-type sequence in the sample, enabling detection of low-abundance amplicons. These amplicons were sequenced directly (Eurofins) and evidence of mutagenesis was conferred by the presence of multiple peaks, representing the different DSB-repair events across the population of cells, visible after the expected cut site (S1 Fig). These chromatogram signals were analysed using the ICE software that determines rates of CRISPR-Cas9 editing at a specific, sgRNA directed genomic location within a cell population (Synthego, https://ice.synthego.com/).

## Results

### Codon optimisation and sgRNA structure have minimal effects on the efficiency of targeted mutagenesis in plants

**Fig 1:**
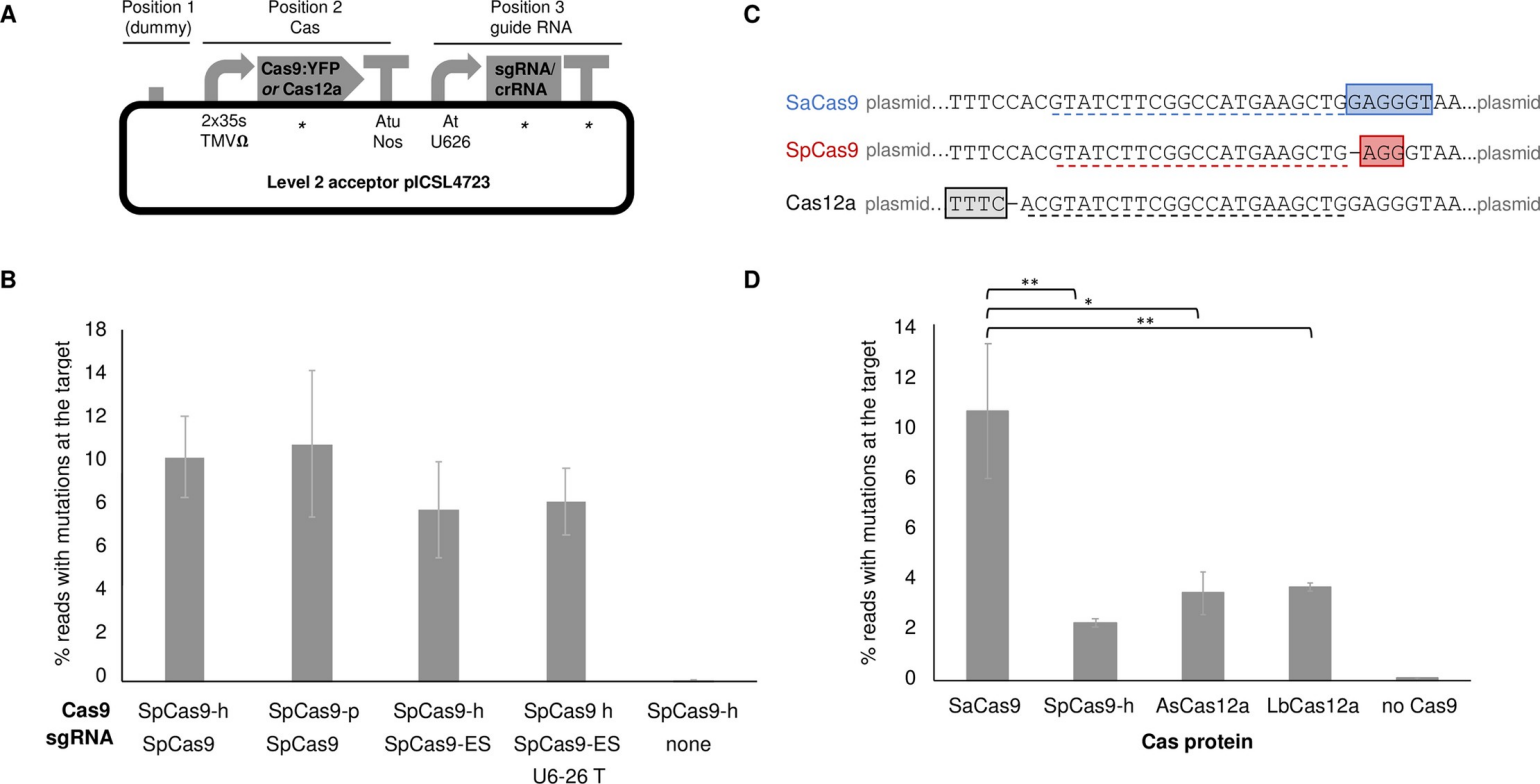
Comparison of Cas9 and Cas12 nucleases at a single target. (A) All constructs were assembled in the same backbone, using the same regulatory elements. Asterisks (*) represent elements compared in this study. The ‘dummy’ consists of 15 random nucleotides and enables the Cas9 and sgRNA expression cassettes to be reassembled with, e.g. a selectable marker cassette, in Position 1. (B) Assessment of codon-optimisation and sgRNA scaffold on the efficiency of Cas9-mediated targeted mutagenesis at *PDS1* in *Nicotiana benthamiana* protoplasts (h = human; p = plant; ES = extended stem; T = terminator). (C) An identical target sequence was used to test Cas proteins from different bacteria. The target was flanked by the preferred cognate protospacer adjacent motif (PAM) for SpCas9 (red box), SaCas9 (blue box) or Cas12a (black box). The spacer sequence used in each sgRNA (Cas9) or crRNA (Cas12a) are indicated by dotted lines. (D) Under identical experimental conditions, SaCas9 induced more mutations at the target than SpCas9, AsCas12a or LbCas12a. Error bars = standard error of the mean; n = 4. Post hoc comparisons using Tukey's Honest Significant Difference test indicted significant differences: * = p<0.05, ** = p<0.01.

Prior to comparison with other Cas nucleases, we first assessed several variables for RNA-guided Cas9. We assessed the effect of codon-optimisation, comparing human (SpCas-h) versus plant (SpCas9-p), as well as variations in the single guide RNA sequence, comparing two sgRNAs: sgRNA and a sgRNA with extended step-loops (sgRNA-ES; [49]. We also compared the use of a previously extended endogenous terminator for sgRNA expression cassettes [50,51]. In these and subsequent experiments, all constructs were assembled similarly using identical regulatory sequences (Fig 1A). All sgRNAs contained a spacer to direct Cas9 to a target in the phytoene desaturate gene of *Nicotiana benthamiana* (*NbPDS1*) and constructs were delivered to protoplasts isolated from leaves of *N*. *benthamiana*. Following a quantitative assessment of the frequency of mutagenesis by Illumina sequencing, we found no significant differences in the number of sequencing reads with mutations at the target (Fig 1B), indicating that neither human codon optimisation, the shorter stems found in the original sgRNA, or the minimal terminator significantly impaired the efficiency of mutagenesis in plants. In all subsequent experiments, we used SpCas9-h together with its original sgRNA as we have had previous success with these sequence in other species [35].

### Cas proteins from different bacterial species show varied efficiencies of targeted mutagenesis at the same target in identical experimental conditions

To enable a direct comparison of the ability of four Cas proteins, SaCas9, SpCas9, AsCas12 and LbCas12a to induce targeted mutations, without the confounding influence of varied efficiency across targets, we designed three variants of a synthetic target such that the same recognition sequence was adjacent to the preferred PAM for each protein (Fig 1C). We then co-delivered the plasmid containing the target with the cognate PAM to protoplasts together with plasmid DNA encoding each of the four nucleases and an appropriate sgRNA (Cas9) or crRNA (Cas12a) with a spacer to the synthetic target. We observed significantly more mutations at the *NbPDS1* target with SaCas9 than with SpCas9-h, AsCas12 or LbCas12a (Fig 1D).

### Efficiency of SpCas9-induced mutagenesis correlates to GC content of the spacer

Analysis of the Arabidopsis genome for potential target sequences identified 3,853,090 potential targets for SpCas9, SaCas9 or Lb/AsCas12a in coding exons (Table 1). Of the 2,695,798 targets recognised by SpCas9, 61,739 (2.29%) have at least one potential off-target (identical or differing at only a single base) also in a coding exon, 7,201 of which differed by a single base in the first three positions. The total number of targets is expectedly lower for SaCas9 and Lb/AsCas12a, which recognise longer PAMs, (Table 1). We filtered these targets for those in genes previously shown to be expressed in leaves (see methods) and selected sequences for functional analysis in Arabidopsis leaf-derived protoplasts.

**Fig 2:**
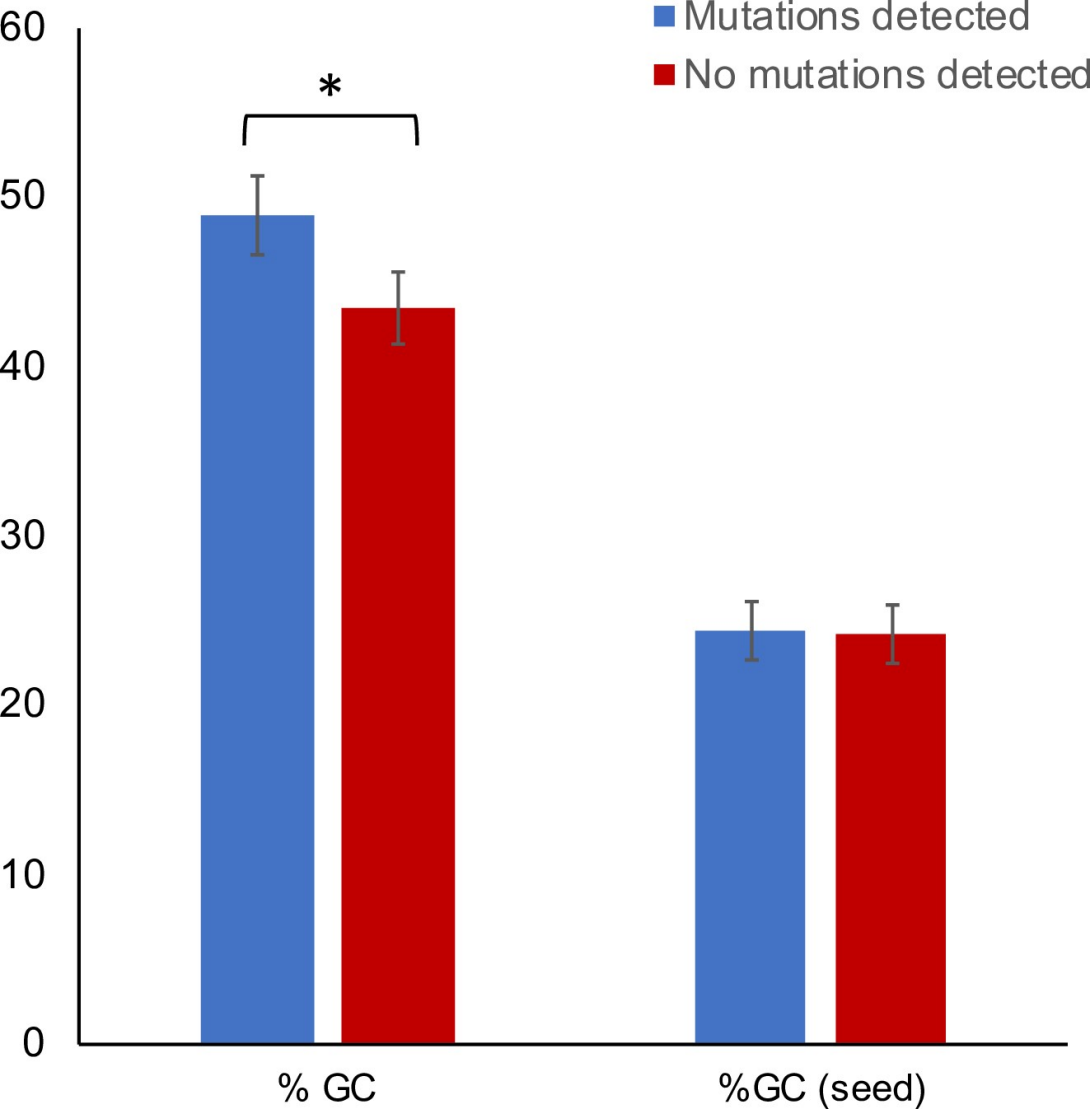
Nucleotide content analysis of 33 spacer sequences. All spacers have cognate targets in the coding sequences of leaf-expressed genes tested in sgRNAs with SpCas9-h. Each spacer was incorporated into a sgRNA and tested in Arabidopsis protoplasts. Blue bars indicate mutations were detected at target; red bars indicate no mutations detected at target. Seed = six base pairs adjacent to PAM. * = p-value 0.044591. The result is significant at p <0.05.

We were able to detect evidence of induced mutations at 14 out of 33 candidate targets with prefect identity to the spacer for SpCas9 (the sequences of all targets tested are provided in S1 Table). We were able to detect evidence of induced mutations at seven out of nine candidate targets with perfect identity to the spacer for SaCas9. The sample set for SaCas9 is too small for meaningful analysis, however, analysis of nucleotide composition of the 33 SpCas9 targets found that the %GC content of spacers used where mutagenesis was detected was significantly greater than those for which no activity was detected (single-tailed T-test, P<0.05) (Fig 2). The data indicates that GC content of spacers should, for maximal efficiency, be greater than 40%. In contrast, we found no differences in the GC content within the seed region.

**Table 1:** Numbers of Cas9 and Cas12 targets and potential off-targets in Arabidopsis coding exons.

Cas nuclease	Target	Total number of targets in coding exons	Number and similarity of potential off-targets in coding exons		
			Identical to target	One SNP in positions 1, 2 or 3 (distal to PAM)	One SNP at any other position
SaCas9	N_21_-NNGGGT	118,048	1,556	228	557
SpCas9	N_20_-NGG	2,695,798	45,707	7,201	16,334
Lb/As Cas12a	TTTV-N_23_	1,039,244	13,644	1,903	13,644

### 'High-fidelity' variants of Cas9 show improved specificity at some targets

**Fig 3:**
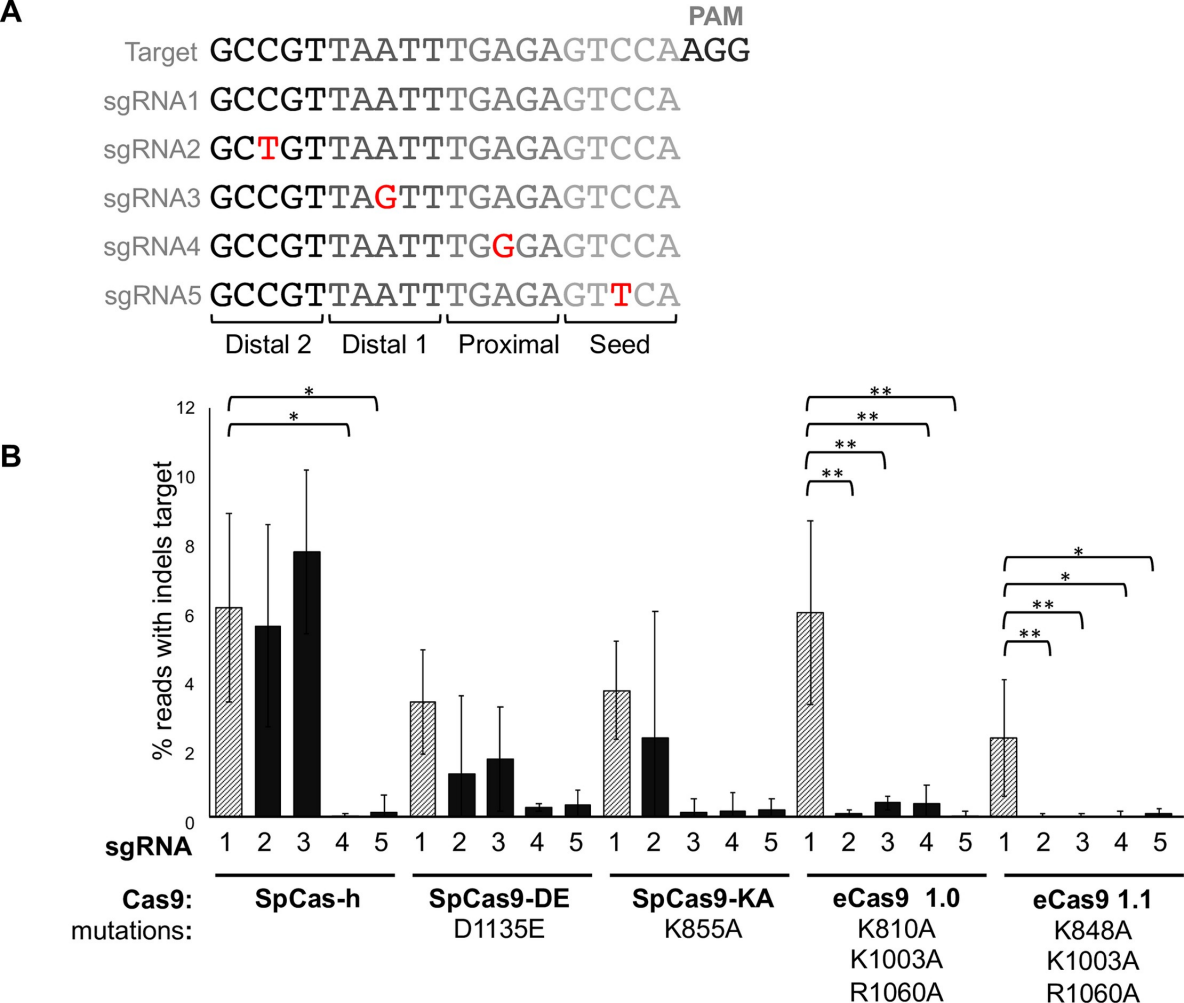
Assessment of Cas9 specificity at an identical target. **(A)** Schematic showing transversions in four regions of the spacer sequences of sgRNAs targeting *NbPDS1* **(B)** The efficiency of targeted mutagenesis is impacted by transversions in the spacer. Shaded bars represent a perfect match of the spacer to the target, black bars show sgRNAs with spacers with transversions relative to the target. Error bars = standard error of the mean; n = 4. Post hoc comparisons using Tukey's Honest Significant Difference test indicted significant differences: * = p<0.05, ** = p<0.01.

To directly compare the efficiency and specificity, *in planta*, of wild-type SpCas9 and the engineered variants, SpCas9 DE [26], SpCas9 KA, eCas9 1.0 and eCas9 1.1 [39], we used a similar experimental process as reported by [26]. We designed a set of five sgRNAs each with a mutation in a different base of a spacer designed to target the *NbPDS* gene (Fig 3A). A sgRNA with an exactly matching spacer, or one of the five variants was delivered to *N*. *benthamiana* protoplasts in combination with each of the five variants of SpCas9 and the number of targeted mutations was quantified using Illumina sequencing. While the number of mutations induced by wild-type Cas9 was significantly reduced when the spacer contained a mutation in the region close to the PAM, the presence of a mismatch between the spacer and target in the distal region had minimal effects (Fig 3B). In contrast, the frequency of mutations induced by the variants eCas9 1.0 and 1.1 was significantly reduced by a mismatch in any region of the sgRNA (Fig 3B). This experiment, although quantitative, was difficult to scale across a larger number of targets. To compare the efficiency of mutagenesis across a larger number of targets and to observe the performance of spacers at non-identical endogenous targets, we conducted an analysis of the Arabidopsis genome to identify pairs of targets that differed by a single base pair. For each target at which mutagenesis was detected using SpCas9-h or SaCas9, the same sgRNAs were also tested with a number of variants of SpCas9 (eCas9 1.0, eCas9 1.1, eSaCas9 [39] and the recently reported xCas9 3.7 [40]) all described to have reduced activity at targets with a less than perfect match to the spacer. In addition to analysis at the targets, we also analysed the identified off-target locus for evidence of mutagenesis (Table 2). Of eight targets at which SpCas9-h was able to induce mutations, the SpCas9 variants were only able to induce mutations at three (xCas9 3.7 and eCas9 1.0) or four (eCas91.1) targets (Table 2). SpCas9-h also induced mutations at all off-targets that differed from the spacer by one nucleotide in the first three positions. Analysis of the sequence chromatogram using the Inference of CRISPR Editing (ICE) software indicated that, in most cases, SpCas9-h induced mutations at a similar efficiency at the off-target as at the target (Table 2 and S1 Fig). In some cases, however, the efficiency of mutagenesis was reduced at off-target loci when the sgRNA was delivered with a variant of Cas9 (Table 2 and S1 Fig). In agreement with the data obtained for *NbPDS* (Fig 3), the efficiency of mutagenesis at some targets was similar for SpCas9-h and eCas9 1.0, while efficiency at the off-target was reduced with eCas9 1.0 (Table 2). However, at several other targets, the SpCas9 variants were unable to induce mutations. In addition, the use of variants did not always reduce mutagenesis at the off-target locus (Table 2).

**Table 2:** Assessment of the specificity of Cas9 variants at targets in Arabidopsis coding sequences.

Gene Model Identifier	Target (bold) and off-targets	Cas9 nuclease			
		SpCas9-h	xCas9 3.7	eCas9 1.0	eCas9 1.1
AT5G58420	**TGGCAAAGGAACAAAGC|CATGGG**	Y (41)	N (0)	Y (37)	Y (15)
AT5G07090	CGGCAAAGGAACAAAGC|CATGGG	Y (67)	N (1)	N (7)	Y (3)
AT2G32730	**GGGAAGTATCAACAAGC|CATGGG**	Y (53)	N (0)	Y (39)	Y (58)
AT1G04810	GGAAAGTATCAACAAGC|CATGGG	Y (39)	N (0)	Y (24)	Y (30)
AT3G14720	**CAGAATGATCCACAGAG|CTCAGG**	Y (55)	Y (35)	Y (48)	N (1)
AT1G53510	AAGAATGATCCACAGAG|CTCAGG	Y (13)	N (0)	Y (18)	N (2)
AT5G10620	**CTGATGATTCAGGACCA|TGGAGG**	Y (24)	Y (23)	Y (32)	N (7)
AT5G10605	ATGATGATTCAGGACCA|TGGAGG	Y (39)	Y (34)	Y (26)	N (0)
AT5G19360	**CCATACTTGCCATTCCA|TGGGGG**	Y (47)	Y (30)	N (1)	Y (36)
AT5G12180	GCATACTTGCCATTCCA|TGGGGG	Y (45)	Y (41)	Y (39)	Y (33)
AT5G04770	**GTCGAGACGACTAGCAC|GGCCGG**	Y*	N*	N*	N*
AT3G10600	GTAGAGACGACTAGCAC|GGCCGG	Y*	N*	N*	N*
AT3G54020	**CGACAGTGATAGTTAGG|GCCAGG**	Y*	N*	N*	N*
AT2G37940	CGGCAGTGATAGTTAGG|GCCAGG	Y*	N*	N*	N*
AT2G68260	**TGAGCCGGCTGAGCCAT|GGAAGG**	Y*	N*	N*	N*
AT1G68260	TGTGCCGGCTGAGCCAT|GGAAGG	Y*	N*	N*	N*
		**SaCas9**	**eSaCas9**		
AT5G04160	**TATGACCTTTAAGAGAGA|AGCTTGGGT**	Y	Y		
AT3G10290	CATGACCTTTAAGAGAGA|AGCTTGGGT	Y	N		
AT1G62600	**GACGCTCGAATGAACAAC|GGATCGGGT**	Y*	Y*		
AT1G63390	AACGCTCGAATGAACAAC|GGATCGGGT	Y*	Y*		
AT1G73460	**TTCATGGTGATCAGGACT|TCCACGGGT**	Y*	N*		
AT1G73450	ATCATGGTGATCAGGACT|TCCACGGGT	Y*	N*		
AT1G14910	**GCCCTTGAAATATACAAG|CGTGCGGGT**	Y*	Y*		
AT2G01600	TCCCTTGAAATATACAAG|CGTGCGGGT	Y*	N*		

Differences between target (bold text) and off-target pairs are shown in red text. The Protospacer Adjacent Motif (PAM) is shown in blue text. Numbers in parentheses are scores from Inference of CRISPR Editing (ICE) software that quantifies the efficiency of induced mutations at the target from the sequence chromatogram. Y/N indicates that mutations at the target were/were not detected

* indicates assessment made by detection of deletion induced with a second sgRNA.

| indicates expected point of cleavage.

We also compared the efficiency and specificity of SaCas9 with an engineered variant eSaCas9 [39]. Of four targets at which SaCas9 was able to induce mutations, eSaCas9 variants were only able to induce mutations at three. In similarity with SpCas9-h, SaCas9 also induced mutations at all off-targets that differed from the spacer by one nucleotide in the first three positions. In contrast, eSaCas9 only induced mutations at one target without perfect identity to the spacer.

## Discussion

Components of bacterial CRISPR/Cas systems have been applied to a wide variety of model and economically-important plant species including dicotyledonous fruit crops such as strawberries [52], tomatoes [53] and oranges [54] as well as monocotyledonous grain crops such as barley [35], wheat [55] and maize [56]. In most cases, engineered plants are regenerated via somatic embryogenesis in tissue culture; a process that is both laborious and time-consuming. Consequently, researchers are interested in the efficiency of the molecular tools they deliver. Since the first reports of Cas9 from *S*. *pyogenes* being applied as tool for genome engineering, there has been much interest in novel properties associated with Cas nucleases from addition CRISPR systems [3,24]. These include comparative efficiency and the potential for engineering at an increased number of genomic targets due to the recognition of additional PAMs. Because of this, Cas nucleases have often, necessarily, used different targets. Together with other differences introduced by different construct designs and experimental conditions, it is difficult to draw conclusions about relative efficiencies. Our experimental strategies allowed us to compare the efficiency of different components for genome engineering on the same target and indicate that SaCas9 may have the highest efficiency of targeted mutagenesis in plants (Fig 1). The drawback of our experimental design, however, is that that the same experimental conditions are unlikely to be optimal for each Cas nuclease. For example, recent work has shown that temperature can increase the activity of Cas12a [33]. It is also important to note that our experimental system, necessarily, used the same regulatory elements in order to compare different nucleases. Since our experiments were performed in leaf-derived protoplasts of *N*. *benthamiana* and *A*. *thaliana*, we utilised the frequently-used 35s promoter from Cauliflower Mosaic Virus (CaMV-35s), which expresses strongly in this cell type, to drive expression of all nucleases. This promoter will not be optimal for all species and the choice of promoters is likely to contribute to efficiency in different species. Even in Arabidopsis, where stable transgenesis utilises a floral-dip method, it is preferential to use a promoter that expressed strongly in developing embryos to minimise the recovery of plants that are genetically-chimeric due to expression of the Cas-nuclease in somatic cells during development [50,57,58].

Much of the work on sequence determinants of spacers performed to date has been performed in mammalian cell cultures [59–63]. These systems provide the advantage of being able to delivery libraries of sgRNAs targeting genes involved in a particular process followed by the selection of cells with the expected phenotype correlating to disruption of those genes to be selected (e.g. by staining and FACS). In an analysis of a number of datasets, including one of 4,000 sgRNA targeting 17 genes, [61] concluded that the patterns of activity are complex and are not likely to be apparent by examining smaller numbers of sgRNA:DNA interactions. Current methods of DNA delivery to plant cells include the preparation of separate strains of *Agrobacterium tumefaciens* for delivery to callus or tissues, or the direct delivery of plasmids either to protoplasts or to tissues (using biolistic delivery). Delivery of a large-scale combined library of constructs using these methods would result in populations of cells with multiple induced mutations from which it would not be possible to identify or separate cells with individual genotypes. In our study, we delivered individual constructs to aliquots of protoplasts prepared from the same batch of leaf tissue. This allowed us to work with larger number of constructs, however, since cultures of protoplasts cannot be perpetuated (they reform cell walls and form masses of cells if allowed or induced to divide), it is not possible to select populations of cells beyond the first 24–48 hours. The advantages of this system, however, allowed us to compare the impact of a number of different components by allowing similar levels of expression across different experiments that are difficult to achieve with integrated transgenes, the expression of which is subject to copy number and the location of integration [64]. Using this method, we attempted to induce mutations at over 45 targets in the Arabidopsis genome, using sgRNAs with spacers with both perfect and imperfect identity. This enabled us to compare the features of functional spacers (those that were able to induce targeted mutations) with non-functional spacer noting a correlation with GC content (Fig 2), broadly in agreement with that reported for mammals [59]. We did not, however, note any determinants for sequence composition in the seed region or at specific bases, likely because of the comparatively small size of our dataset. The correlation of efficiency with GC content may be related to nucleosome occupancy, previously reported to influence the ability of Cas9 to recognise its targets [65]; nucleosome-depleted regions in Arabidopsis tend to have a higher GC content [66]. As this negative correlation between nucleosome occupancy and GC content is not known to be common across all organisms, further studies in a wider range of plant species will need to be performed to determine if efficiency can be predicted by GC content alone.

Analysis of Arabidopsis coding sequences revealed that many targets for Cas-mediated genome engineering have one or more potential off-targets in other coding sequences that differ by just a single base (Tables 1 and S1). We provide experimental evidence that SpCas9 and SaCas9 are readily able to induce mutations at targets with less than perfect identity to the spacer (Table 2 and Fig 3). Different laboratories have engineered variants of SpCas9 and SaCas9, reportedly reducing their activity at targets to which the spacer does not have 100% identity [40,41,67,68]. To compare these proteins and determine their function in plants, we measured their efficiency and specificity across a number of targets (Table 2 and Fig 3). Previous reports have found that the engineered variants showed increased specificity and retained a similar efficiency as the wild-type protein when the spacer was identical to the target [68]. We found this to be true at some targets but at others the engineered variants were often either less efficient or non-functional. Additionally, we found that use of the variants did not always increase specificity and that the efficiency of mutagenesis at some off-targets was similar to wild-type Cas9.

**Fig 4:**
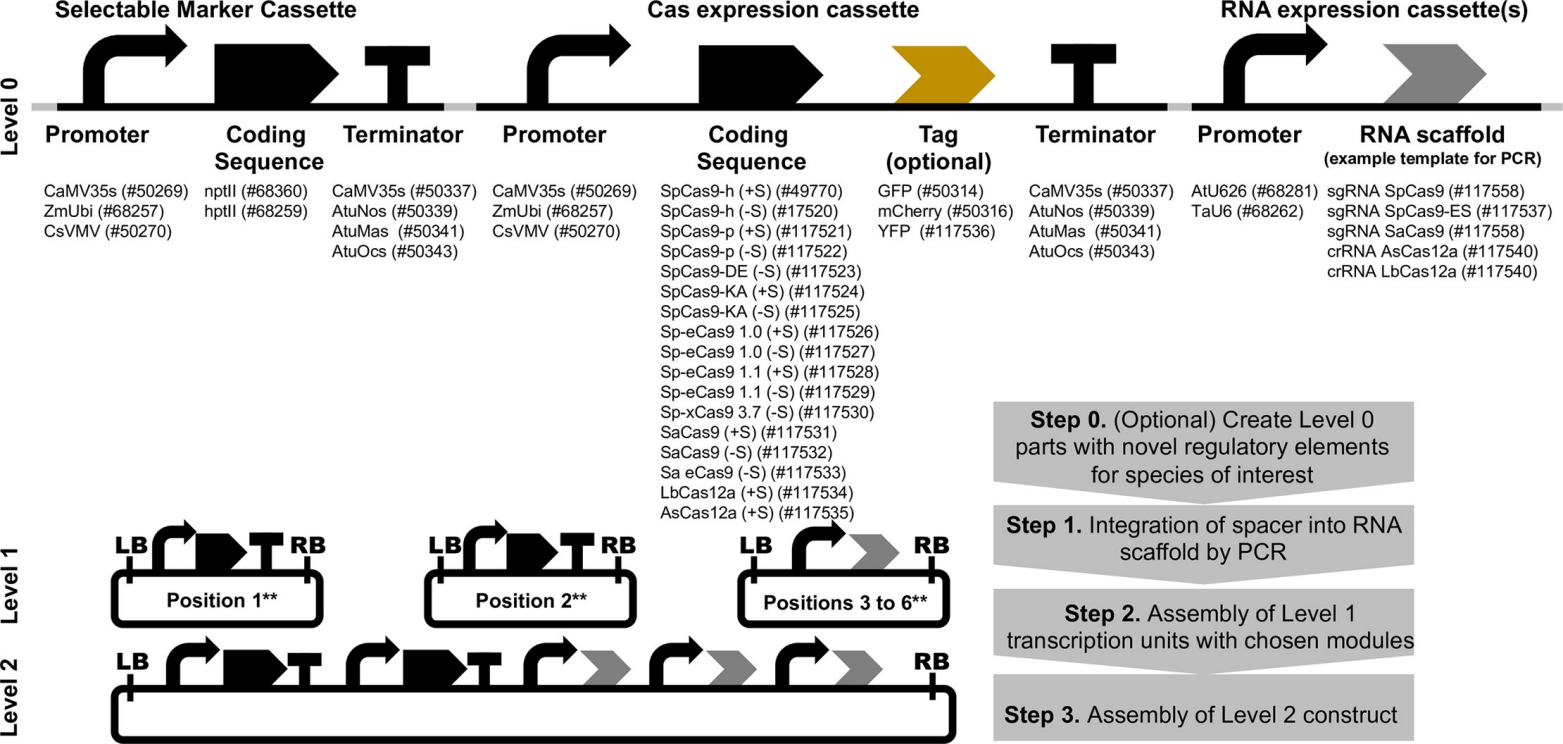
An expanded toolkit for Cas-mediated genome engineering in plants. Regulatory elements and coding sequences are cloned as Level 0 parts, enabling one-step assembly into transcriptional units mediated by *BsaI* (e.g. using the Plant MoClo Toolkit, [45]) and subsequently into multigene constructs. Numbers in parentheses represent catalogue number at Addgene. **For simplicity, all cassettes are shown assembled on the forward strand. However, the orientation of any cassette in the final construct can be altered by use of a reverse Level 1 acceptor. ‘+S’ = with stop codon ‘-S’ = no stop codon.

To perform these experiments, we utilised standardised modular cloning techniques to create a suite of comparable constructs to enable direct comparison of multiple different tools for genome engineering. Type IIS mediated assembly methods have been widely utilised to facilitate the construction of the complex plasmids required for multiplexed genome editing [11,29–31]. In addition to standardising our experimental process, we are able to provide an expanded toolkit of modular, reusable parts for plant genome engineering that will facilitate their application in new studies (Fig 4). The basic (Level 0) parts are flanked with inverted *BsaI* sites that will release parts with overhangs in the common genetic syntax for plants [46], making them amenable for reuse with a number of different assembly toolkits for plants.

This study has provided data that will be useful to plant scientists when selecting targets and molecular components for Cas-mediated targeted mutagenesis in plants. We found that SaCas9 is likely to be the most efficient nuclease. When selecting targets for mutagenesis with SpCas9, spacers with a GC content above 45% may be preferential. Regarding specificity, both SaCas9 and SpCas9 are highly likely to induce mutations at any off-target loci that differ from the target by a single nucleotide close to the 5' end. Specificity may be improved by the use of eCas9 1.0, eCas9 1.1 or xCas9 3.7, however, a greater number of targets should be tested as efficiency is likely to be reduced or eliminated at some targets.

## Supporting information

S1 Table Genomic targets for Cas nucleases in *Arabidopsis thaliana* coding exons. (XLSX)

S2 Table Details of plasmid constructs used in this study. (XLSX)

S3 Table Oligonucleotide primers used to amplify genomic targets. (PDF)

S1 Fig Chromatograms and gel images of Cas-induced targeted mutations and deletions. (PDF)

## References

[1] Mali P, Yang L, Esvelt KM, Aach J, Guell M, DiCarlo JE, et al RNA-Guided Human Genome Engineering via Cas9 Prashant. Science. 2013;339(6121):823–6. 10.1126/science.1232033 23287722 PMC3712628

[2] Cong L, Ran FA, Cox D, Lin S, Barretto R, Habib N, et al Multiplex genome engineering using CRISPR/Cas systems. Science 2013;339(6121):819–23. 10.1126/science.1231143 23287718 PMC3795411

[3] Zetsche B, Gootenberg JS, Abudayyeh OO, Regev A, Koonin E V., Zhang F, et al Cpf1 Is a Single RNA-Guided Endonuclease of a Class 2 CRISPR-Cas System. Cell 2015;163(3):759–71. 10.1016/j.cell.2015.09.038 26422227 PMC4638220

[4] Yin K, Gao C, Qiu J-L. Progress and prospects in plant genome editing. Nat Plants. 2017;3:17107 10.1038/nplants.2017.107 28758991

[5] Gao C. The future of CRISPR technologies in agriculture. Nature Reviews Molecular Cell Biology. 2018; 19:275–276 10.1038/nrm.2018.2 29382940

[6] Ricroch A, Clairand P, Harwood W. Use of CRISPR systems in plant genome editing: toward new opportunities in agriculture. Emerg Top Life Sci. 2017;1(2):168–92. 10.1042/ETLS20170085 PMC7288993 33525765

[7] Barrangou R, Fremaux C, Deveau H, Richards M, Boyaval P, Moineau S, et al CRISPR provides acquired resistance against viruses in prokaryotes. Science 2007;315(5819):1709–12. 10.1126/science.1138140 17379808

[8] Sternberg SH, Redding S, Jinek M, Greene EC, Doudna JA. DNA interrogation by the CRISPR RNA-guided endonuclease Cas9. Nature. Nature Publishing Group; 2014;507(7490):62–7. 10.1038/nature13011 24476820 PMC4106473

[9] Schiml S, Puchta H. Revolutionizing plant biology: multiple ways of genome engineering by CRISPR/Cas. Plant Methods. BioMed Central; 2016;12(1):8. 10.1186/s13007-016-0103-0 PMC4730597 26823677

[10] Hu Z, Cools T, De Veylder L. Mechanisms Used by Plants to Cope with DNA Damage. Annu Rev Plant Biol. 2016;67(1):439–62. 26653616 10.1146/annurev-arplant-043015-111902

[11] Cermak T, Curtin S, Gil-Humanes J, Cegan R, Starker CG, Kono TJY, et al A multi-purpose toolkit to enable advanced genome engineering in plants. Plant Cell. 2017; 10.1105/tpc.16.00922 PMC5502448 28522548

[12] Gil-Humanes J, Wang Y, Liang Z, Shan Q, Ozuna C V., Sanchez-Leon S, et al High-efficiency gene targeting in hexaploid wheat using DNA replicons and CRISPR/Cas9. Plant J. 2017;89(6):1251–62. 10.1111/tpj.13446 27943461 PMC8439346

[13] Baltes NJ, Gil-humanes J, Cermak T, Atkins PA, Voytas DF. DNA Replicons for Plant Genome Engineering. Plant Cell. 2014;26(1):151–63. 10.1105/tpc.113.119792 24443519 PMC3963565

[14] Begemann MB, Gray BN, January E, Gordon GC, He Y, Liu H, et al Precise insertion and guided editing of higher plant genomes using Cpf1 CRISPR nucleases. Sci Rep. Springer US; 2017;7(1):1–6. 10.1038/s41598-017-11760-6 PMC5599503 28912524

[15] Li J-F, Norville JE, Aach J, McCormack M, Zhang D, Bush J, et al Multiplex and homologous recombination–mediated genome editing in Arabidopsis and Nicotiana benthamiana using guide RNA and Cas9. Nat Biotechnol. 2013, 8 8;31(8):688–91. 10.1038/nbt.2654 23929339 PMC4078740

[16] Čermák T, Baltes NJ, Čegan R, Zhang Y, Voytas DF. High-frequency, precise modification of the tomato genome. Genome Biol. 2015;16(1):232. 26541286 10.1186/s13059-015-0796-9 PMC4635538

[17] Kaya H, Mikami M, Endo A, Endo M, Toki S. Highly specific targeted mutagenesis in plants using Staphylococcus aureus Cas9. Sci Rep. 2016;6(1):26871. 27226350 10.1038/srep26871 PMC4881040

[18] Steinert J, Schiml S, Fauser F, Puchta H. Highly efficient heritable plant genome engineering using Cas9 orthologues from Streptococcus thermophilus and Staphylococcus aureus. Plant J. 2015;84(6):1295–305. 10.1111/tpj.13078 26576927

[19] Ran FA, Cong L, Yan WX, Scott DA, Gootenberg JS, Kriz AJ, et al In vivo genome editing using Staphylococcus aureus Cas9. Nature. 2015;520(7546):186–91. 10.1038/nature14299 25830891 PMC4393360

[20] Xu R, Qin R, Li H, Li D, Li L, Wei P, et al Generation of targeted mutant rice using a CRISPR-Cpf1 system. Plant Biotechnol J. 2017;15(6):713–7. 10.1111/pbi.12669 27875019 PMC5425385

[21] Tang X, Lowder LG, Zhang T, Malzahn AA, Zheng X, Voytas DF, et al A CRISPR–Cpf1 system for efficient genome editing and transcriptional repression in plants. Nat Plants. 2017;3:17018 10.1038/nplants.2017.18 28211909

[22] Kleinstiver BP, Prew MS, Tsai SQ, Nguyen NT, Topkar V V., Zheng Z, et al Broadening the targeting range of Staphylococcus aureus CRISPR-Cas9 by modifying PAM recognition. Nat Biotechnol. 2015;33(12):1293–8. 10.1038/nbt.3404 26524662 PMC4689141

[23] Xie H, Tang L, He X, Liu X, Zhou C, Liu J, et al SaCas9 Requires 5′-NNGRRT-3′ PAM for Sufficient Cleavage and Possesses Higher Cleavage Activity than SpCas9 or FnCpf1 in Human Cells. Biotechnol J. 2018;14(4):e1700561. 10.1002/biot.201700561 29247600

[24] Friedland AE, Baral R, Singhal P, Loveluck K, Shen S, Sanchez M, et al Characterization of Staphylococcus aureus Cas9: A smaller Cas9 for all-in-one adeno-associated virus delivery and paired nickase applications. Genome Biol. 2015;24(16):257. 10.1186/s13059-015-0817-8 PMC4657203 26596280

[25] Yamano T, Zetsche B, Ishitani R, Zhang F, Nishimasu H, Nureki O. Structural Basis for the Canonical and Non-canonical PAM Recognition by CRISPR-Cpf1. Mol Cell. 2017;67(4):633–45. 10.1016/j.molcel.2017.06.035 28781234 PMC5957536

[26] Kleinstiver BP, Prew MS, Tsai SQ, Topkar V V., Nguyen NT, Zheng Z, et al Engineered CRISPR-Cas9 nucleases with altered PAM specificities. Nature. 2015;523(7561):481–5. 10.1038/nature14592 26098369 PMC4540238

[27] Werner S, Engler C, Weber E, Gruetzner R, Marillonnet S. Fast track assembly of multigene constructs using Golden Gate cloning and the MoClo system. Bioeng Bugs. 2012;3(1):38–43. 10.1371/journal.pone.0016765 22126803

[28] Engler C, Kandzia R, Marillonnet S. A one pot, one step, precision cloning method with high throughput capability. PLoS One. 2008;3(11):e3647 10.1371/journal.pone.0003647 18985154 PMC2574415

[29] Xing H-L, Dong L, Wang Z-P, Zhang H-Y, Han C-Y, Liu B, et al A CRISPR/Cas9 toolkit for multiplex genome editing in plants. BMC Plant Bio. 2014;14(1):327. 25432517 10.1186/s12870-014-0327-y PMC4262988

[30] Ma X, Zhang Q, Zhu Q, Liu W, Chen Y, Qiu R, et al A Robust CRISPR/Cas9 System for Convenient, High-Efficiency Multiplex Genome Editing in Monocot and Dicot Plants. Mol Plant. 2015;8(8):1274–84. 10.1016/j.molp.2015.04.007 25917172

[31] Vazquez-Vilar M, Bernabé-Orts JM, Fernandez-del-Carmen A, Ziarsolo P, Blanca J, Granell A, et al A modular toolbox for gRNA–Cas9 genome engineering in plants based on the GoldenBraid standard. Plant Methods. 2016;12(1):10. 26839579 10.1186/s13007-016-0101-2 PMC4736081

[32] Lee K, Zhang Y, Kleinstiver BP, Guo JA, Aryee MJ, Miller J, et al Activities and specificities of CRISPR/Cas9 and Cas12a nucleases for targeted mutagenesis in maize. Plant Biotechnol J. 2018;1–11. 10.1111/pbi.12982 PMC6320322 29972722

[33] Moreno-Mateos MA, Fernandez JP, Rouet R, Vejnar CE, Lane MA, Mis E, et al CRISPR-Cpf1 mediates efficient homology-directed repair and temperature-controlled genome editing. Nat Commun. 2017;8(8):2024. 29222508 10.1038/s41467-017-01836-2 PMC5722943

[34] Li Z, Liu Z, Xing A, Moon BP, Koellhoffer JP, Huang L, et al Cas9-Guide RNA Directed Genome Editing in Soybean. Plant Physiol. 2015;169(2):960–70. 10.1104/pp.15.00783 26294043 PMC4587461

[35] Lawrenson T, Shorinola O, Stacey N, Li C, Østergaard L, Patron NJ, et al Induction of targeted, heritable mutations in barley and Brassica oleracea using RNA-guided Cas9 nuclease. Genome Biology. 2015;30(16):258. 10.1186/s13059-015-0826-7 PMC4663725 26616834

[36] Zhang Q, Xing HL, Wang ZP, Zhang HY, Yang F, Wang XC, et al Potential high-frequency off-target mutagenesis induced by CRISPR/Cas9 in Arabidopsis and its prevention. Plant Mol Biol. 2018;96(4–5):445–56. 10.1007/s11103-018-0709-x 29476306 PMC5978904

[37] Zhang D, Wang Z, Wang N, Gao Y, Liu Y, Wu Y, et al Tissue culture-induced heritable genomic variation in rice, and their phenotypic implications. PLoS One. 2014;9(5):e96879 10.1371/journal.pone.0096879 24804838 PMC4013045

[38] Phillips RL, Kaeppler SM, Olhoft P. Genetic instability of plant tissue cultures: breakdown of normal controls. Proc Natl Acad Sci U S A. 1994;91(12):5222–6. 8202472 10.1073/pnas.91.12.5222 PMC43966

[39] Slaymaker IM, Gao L, Zetsche B, Scott DA, Yan WX, Zhang F. Rationally engineered Cas9 nucleases with improved specificity. Science. 2016;351(6268):84–8. 10.1126/science.aad5227 26628643 PMC4714946

[40] Hu JH, Miller SM, Geurts MH, Tang W, Chen L, Sun N, et al Evolved Cas9 variants with broad PAM compatibility and high DNA specificity. Nature. 2018;556(7699):57–63. 10.1038/nature26155 29512652 PMC5951633

[41] Kleinstiver BP, Pattanayak V, Prew MS, Tsai SQ, Nguyen NT, Zheng Z, et al High-fidelity CRISPR–Cas9 nucleases with no detectable genome-wide off-target effects. Nature. 2016;529(7587):490–5. 10.1038/nature16526 26735016 PMC4851738

[42] Marçais G, Kingsford C. A fast, lock-free approach for efficient parallel counting of occurrences of k-mers. Bioinformatics. 2011;27(6):764–70. 10.1093/bioinformatics/btr011 21217122 PMC3051319

[43] Quinlan AR, Hall IM. BEDTools: A flexible suite of utilities for comparing genomic features. Bioinformatics. 2010;26(6):841–2. 10.1093/bioinformatics/btq033 20110278 PMC2832824

[44] Köster J, Rahmann S. Snakemake-a scalable bioinformatics workflow engine. Bioinformatics. 2012;28(19):2520–2522. 10.1093/bioinformatics/bts480 22908215

[45] Engler C, Youles M, Grüetzner R. A Golden Gate modular cloning toolbox for plants. ACS Synth Biol. 2014;3(11):839–43. 10.1021/sb4001504 24933124

[46] Patron NNJ, Orzaez D, Marillonnet S, Warzecha W, Matthewman C, Youles M, et al Standards for Plant Synthetic Biology: A Common Syntax for Exchange of DNA Parts. New Phytol. 2015;208(1):13–9. 10.1111/nph.13532 26171760

[47] Yoo SD, Cho YH, Sheen J. Arabidopsis mesophyll protoplasts: A versatile cell system for transient gene expression analysis. Nat Protoc. 2007;2(7):1565–72. 10.1038/nprot.2007.199 17585298

[48] Pinello L, Canver MC, Hoban MD, Orkin SH, Kohn DB, Bauer DE, et al Analyzing CRISPR genome-editing experiments with CRISPResso. Nat Biotechnol. 2016;34(7):695–7. 10.1038/nbt.3583 27404874 PMC5242601

[49] Chen B, Gilbert L a, Cimini B a, Schnitzbauer J, Zhang W, Li G-W, et al Dynamic imaging of genomic loci in living human cells by an optimized CRISPR/Cas system. Cell. 2013;155(7):1479–91. 10.1016/j.cell.2013.12.001 24360272 PMC3918502

[50] Wang Z-P, Xing H, Dong L, Zhang H, Han C, Wang X, et al Egg cell-specific promoter-controlled CRISPR/Cas9 efficiently generates homozygous mutants for multiple target genes in Arabidopsis in a single generation. Genome Biology; 2015;16(1):144. 26193878 10.1186/s13059-015-0715-0 PMC4507317

[51] Xu R, Li H, Qin R, Wang L, Li L, Wei P, et al Gene targeting using the Agrobacterium tumefaciens-mediated CRISPR-Cas system in rice. Rice. 2014;7(1):5 10.1186/s12284-014-0005-6 24920971 PMC4052633

[52] Zhou J, Wang G, Liu Z. Efficient genome editing of wild strawberry genes, vector development and validation. Plant Biotechnology Journal. 2018; 16(11)1868–1877. 10.1111/pbi.12922 29577545 PMC6181217

[53] Brooks C, Nekrasov V, Lippman ZB, Van Eck J, Eck J Van. Efficient Gene Editing in Tomato in the First Generation Using the Clustered Regularly Interspaced Short Palindromic Repeats/CRISPR-Associated9 System. Plant Physiol. 2014;166(3):1292–7. 10.1104/pp.114.247577 25225186 PMC4226363

[54] Jia H, Wang N. Targeted genome editing of sweet orange using Cas9/sgRNA. PLoS One. 2014;9(4):e93806 10.1371/journal.pone.0093806 24710347 PMC3977896

[55] Liang Z, Chen K, Zhang Y, Liu J, Yin K, Qiu JL, et al Genome editing of bread wheat using biolistic delivery of CRISPR/Cas9 in vitro transcripts or ribonucleoproteins. Nat Protoc. 2018;13(3):413–30. 10.1038/nprot.2017.145 29388938

[56] Char SN, Neelakandan AK, Nahampun H, Frame B, Main M, Spalding MH, et al An Agrobacterium-delivered CRISPR/Cas9 system for high-frequency targeted mutagenesis in maize. Plant Biotechnol J. 2017;15(2):257–68. 10.1111/pbi.12611 27510362 PMC5259581

[57] Yan L, Wei S, Wu Y, Hu R, Li H, Yang W, et al High-Efficiency Genome Editing in Arabidopsis Using YAO Promoter-Driven CRISPR/Cas9 System. Mol Plant. 2015;8(12):1820–3. 10.1016/j.molp.2015.10.004 26524930

[58] Xu P, Su H, Chen W, Lu P. The Application of a Meiocyte-Specific CRISPR/Cas9 (MSC) System and a Suicide-MSC System in Generating Inheritable and Stable Mutations in Arabidopsis. Front Plant Sci. 2018;9(7):1–12. 10.3389/fpls.2018.00001 30061908 PMC6055057

[59] Doench JG, Hartenian E, Graham DB, Tothova Z, Hegde M, Smith I, et al Rational design of highly active sgRNAs for CRISPR-Cas9–mediated gene inactivation. Nat Biotechnol. 2014 9 3;32:1262–7. 10.1038/nbt.3026 25184501 PMC4262738

[60] Xu H, Xiao T, Chen CH, Li W, Meyer CA, Wu Q, et al Sequence determinants of improved CRISPR sgRNA design. Genome Res. 2015;25(8):1147–57. 10.1101/gr.191452.115 26063738 PMC4509999

[61] Doench JG, Fusi N, Sullender M, Hegde M, Vaimberg EW, Donovan KF, et al Optimized sgRNA design to maximize activity and minimize off-target effects of CRISPR-Cas9. Nat Biotechnol. 2016;34(2):184–91. 10.1038/nbt.3437 26780180 PMC4744125

[62] Gagnon JA, Valen E, Thyme SB, Huang P, Ahkmetova L, Pauli A, et al Efficient mutagenesis by Cas9 protein-mediated oligonucleotide insertion and large-scale assessment of single-guide RNAs. PLoS One. 2014;9(8):e106396. 10.1371/journal.pone.0098186 PMC4038517 24873830

[63] Wang Y, Liu KI, Sutrisnoh NAB, Srinivasan H, Zhang J, Li J, et al Systematic evaluation of CRISPR-Cas systems reveals design principles for genome editing in human cells. Genome Biol. 2018;19(1):62 10.1186/s13059-018-1445-x 29843790 PMC5972437

[64] Kohli A, González-Melendi P, Abranches R, Capell T, Stoger E, Christou P. The quest to understand the basis and mechanisms that control expression of introduced transgenes in crop plants. Plant Signaling and Behavior. 2006;1(4):185–95. 19521484 10.4161/psb.1.4.3195 PMC2634025

[65] Horlbeck MA, Witkowsky LB, Guglielmi B, Replogle JM, Gilbert LA, Villalta JE, et al Nucleosomes impede cas9 access to DNA in vivo and in vitro. Elife. 2016;5:e12677 10.7554/eLife.12677 26987018 PMC4861601

[66] Liu M-J, Seddon AE, Tsai ZT-Y, Major IT, Floer M, Howe GA, et al Determinants of nucleosome positioning and their influence on plant gene expression. Genome Res. 2015;25:1182–95. 10.1101/gr.188680.114 26063739 PMC4510002

[67] Slaymaker IM, Gao L, Zetsche B, Scott DA, Yan WX, Zhang F. Rationally engineered Cas9 nucleases with improved specificity. Science. 2015;10(6268):1126. 10.1126/science.aad5227 PMC4714946 26628643

[68] Zhang D, Zhang H, Li T, Chen K, Qiu JL, Gao C. Perfectly matched 20-nucleotide guide RNA sequences enable robust genome editing using high-fidelity SpCas9 nucleases. Genome Biol. 2017;18(1):191 10.1186/s13059-017-1325-9 29020979 PMC5637269

