# Supplementary material for: Comparison of efficiency and specificity of CRISPR-associated (Cas) nucleases in plants: An expanded toolkit for precision genome engineering
Source: PLoS One. 2019 Feb 27;14(2):e0211598. doi: 10.1371/journal.pone.0211598 (PMC6392405; doi:10.1371/journal.pone.0211598)
Supplement: S3 Table — (PDF) [file pone.0211598.s003.pdf]

S3 Table

Oligonucleotide primers used to amplify genomic targets

[Underline denotes extensions for addition of adapters for preparation of Illumina libraries]

Name	Arabidopsis Gene Model Identifier	Forward primer 5'-3'	Reverse primer 5'-3'
NbPDS – phytoene desturase (*Nicotiana benthamiana*)	N/A	TCGTCGGCAGCGTCAGATGTGTATAAGAGACAGGATAAGGAATTTTGCATAGT	GTCTCGTGGGCTCGGAGATGTGTATAAGAGACAGAGGATTAAAGTCCTTTGTCA
AtADH1 – alcohol dehydrogenase	AT1G77120	TCGTCGGCAGCGTCAGATGTGTATAAGAGACAGTTCTCTCTGTCACACCGATG	GTCTCGTGGGCTCGGAGATGTGTATAAGAGACAGTGGCTGAAGATCAGTCACTC
RPS4A family protein	AT5G58420	TGTGATTAAAAACCGTGAGA	CAAGAGAGGACACAATGGATA
RPS4A family protein	AT5G07090	CTTTGAGACAATCCACATTC	TTGAAATCCAACAACATAAGA
RPS4A family protein	AT5G37072	TTCAAAAAATCAGGAGACTGG	AAGACTTCTAAGTCATATAGAACA
Rpn2/Psmd1 subunit	AT2G32730	TGTTGAAAGAATGCTTGGAA	CATGTCTGTACTCTCTGCGGT
Rpn2/Psmd1 subunit	AT1G04810	TGTTGAACGAATGCTAGACA	CATGTCTATATTCTCTTTGAT
TPR3-related	AT5G27030	GTTCTCTTGTGGAACGAGTA	TAATGAGTGATTTTACCAGCAT
AtMPK19	AT3G14720	TCTGTGACTTTGGACTTGCAA	CATGTGTGGGTGGTTCATTCTA
AtMPK18	AT1G53510	TTGTGACTTTGGATTGGCTA	GGCACAACTCTATACTTATACAAG
ZFN3	AT5G16540	GTAAATTTGATCACCCGATG	GGCGAATCAGATTAGATCAGA
methyltransferase	AT5G10620	AGAAACTGTCTTTATGATTGATAA	GCAGTTTCTTTGTACACGATAG
methyltransferase	AT5G10605	AAGCTCTCAACAGGAAATCA	ACTTTCGACTTACAGTAACAGT
AGL104	AT1G22130	AAGTTTGTAGGTTACAACAACA	CATAGTAGATGCTTCATACGA
AtCPK34	AT5G19360	TGTGGAGCTTAAAGGAGCGT	TCTGGTCCATACTTCCTCCT
CPK17	AT5G12180	TGTGGAGCTTAAAGGGGCTT	GTCACGCAGGGTTTACAACG
UUAT1	AT5G04160	GCATCGTCTTTTGTGCTTCCG	TTAAGAGAATTGAGAACAGAACA
Nucleotide-sugar transporter family protein	AT3G10290	CATTGTCTTTTGTGCCTCTG	TTTCATTACGAGAATTGTAGAGAA
Hypothetical protein	AT1G68250	CTCTCCATATCACTCTTCAACTA	TACATAATTACCTTTTCCGTTGCT
AtCas6	AT5G04770	CAACAATGGTGGTCACTCTT	TTAAAATTTTCAGAAAACCGTG
CAT7	AT3G10600	ATACCGGAACCACGACGGAGACACCT	ATAAACGCCGGAAACTCGCCTGTA
AtIPCS1	AT3G54020	CGCCTTCTTAGTTGTAAGGACTCT	TTACAAGCTATTATTTTGTCCGG
ATIPCS2	AT2G37940	GCATTCTTAGTTGTAAGTTCCTGC	TGTGATTTACAGAGAAGCCT
Flavin-binding monooxygenase family protein	AT1G62600	AGTATATAGACCATGTACAATTTC	TTTCTTCTCTTTCTCCGTAGATT
flavin containing monooxygenase FMO GS-OX-like protein	AT1G63390	AGTATATAGATTATGTGAAATTCC	CAAACCGAATCATCTCCTCGA
flavin containing monooxygenase FMO GS-OX-like protein	AT1G62620	AGTATATAGACCATGTACAATTTC	TTTCTTCTCTTTCTCCGTAGATT
flavin containing monooxygenase FMO GS-OX-like protein	AT1G63370	AGTATATAGACCATGTACAATTTC	GGCACCTGCATTATCACCAA
Protein kinase superfamily protein	AT1G73460	TCAGCGAACTGGAGAGAGTG	CTGCCAAACAGGCTCATCAC
Protein kinase superfamily protein	AT1G73450	TCAGAGAACTGGAAAGAGCG	CTGCCAAACAGGCTCAGTCC
ENTH/ANT	AT1G14910	TCCTTACTGCTACATTCACG	GTAACAAACAAGTTATTATCTGAT
ENTH/ANT	AT2G01600	TTAGTGTTTCTCCTTCATTTGTG	TCAAAGTTGTCCACAGTTAGT
