# Supplementary material for: Comparison of efficiency and specificity of CRISPR-associated (Cas) nucleases in plants: An expanded toolkit for precision genome engineering
Source: PLoS One. 2019 Feb 27;14(2):e0211598. doi: 10.1371/journal.pone.0211598 (PMC6392405; doi:10.1371/journal.pone.0211598)
Supplement: S1 Fig — (PDF) [file pone.0211598.s004.pdf]

S1 Fig. Chromatograms and gel images of Cas-induced targeted mutations and deletions. For each target the PAM is shown in blue underline and for each candidate off-target mismatches to the spacer are shown in red. The expected cleavage site is marked with a vertical red line (I) in the target and with a red arrow ▲ in the chromatogram.

AT5G58420

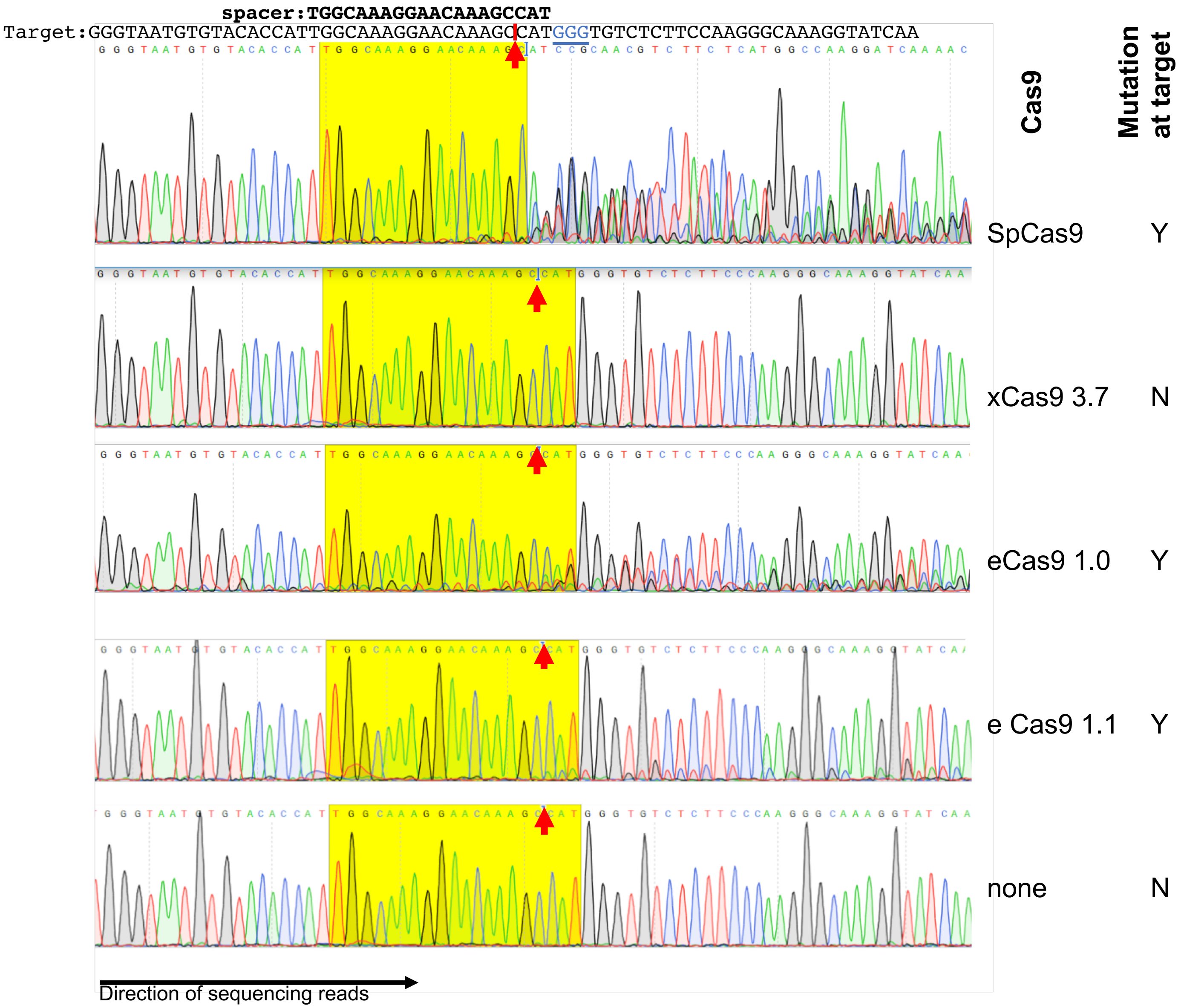
spacer:TGGCAAAGGAACAAAGCCAT
Target:GGGTAATGTGTACACCATTGGCAAAGGAACAAAGCCATGGGTGTCTCTTCCAAGGGCAAAGGTATCAA
Cas9
Mutation at target
SpCas9 Y
xCas9 3.7 N
eCas9 1.0 Y
e Cas9 1.1 Y
none N
Direction of sequencing reads

AT5G07090

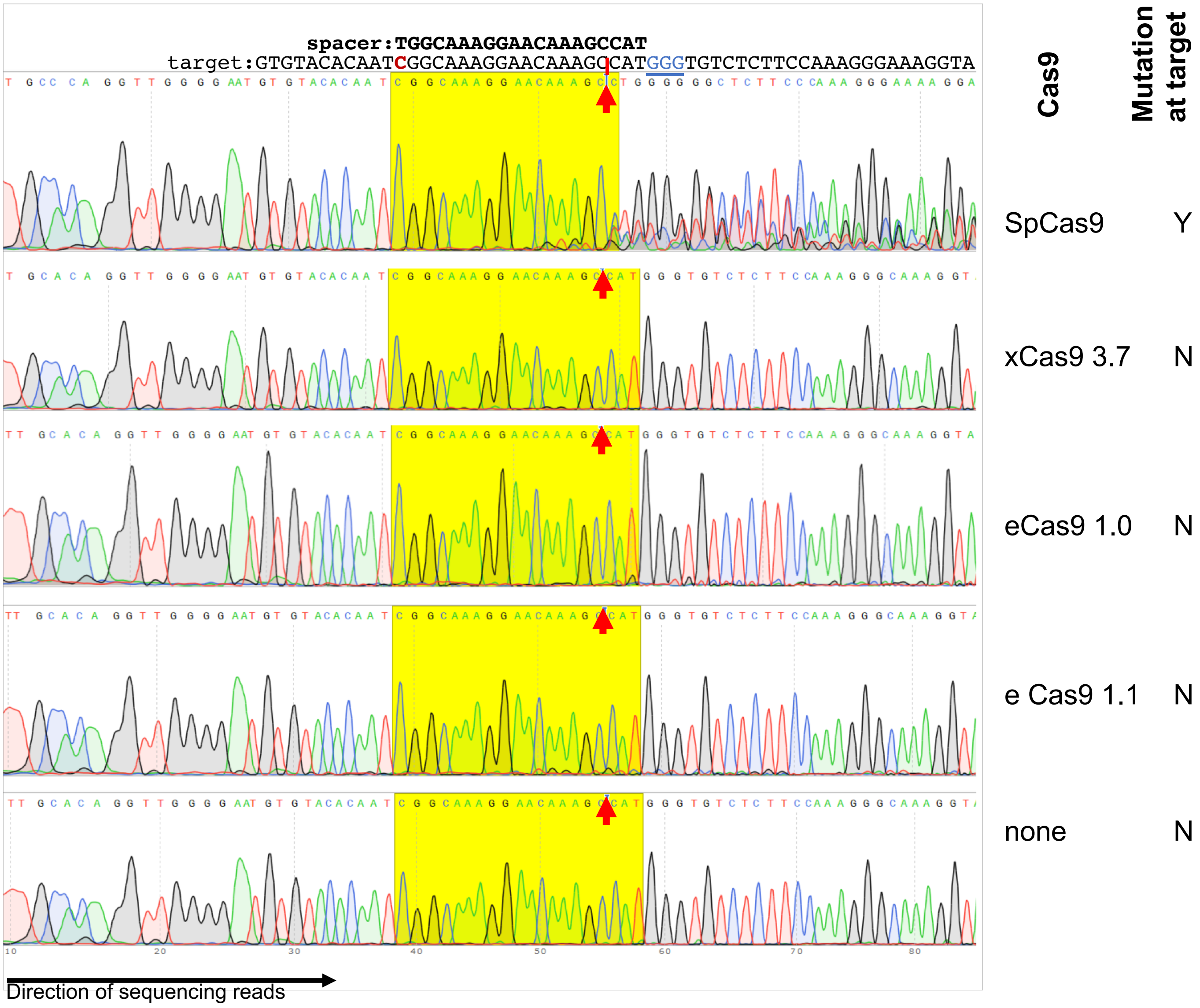
spacer:TGGCAAAGGAACAAAGCCAT
target:GTGTACACAATCGGCAAAGGAACAAAGCCATGGGTGTCTCTTCCAAAGGGAAAGGTA
Cas9
Mutation at target
SpCas9
Y
xCas9 3.7
N
eCas9 1.0
N
e Cas9 1.1
N
none
N
Direction of sequencing reads

AT5G37072 b

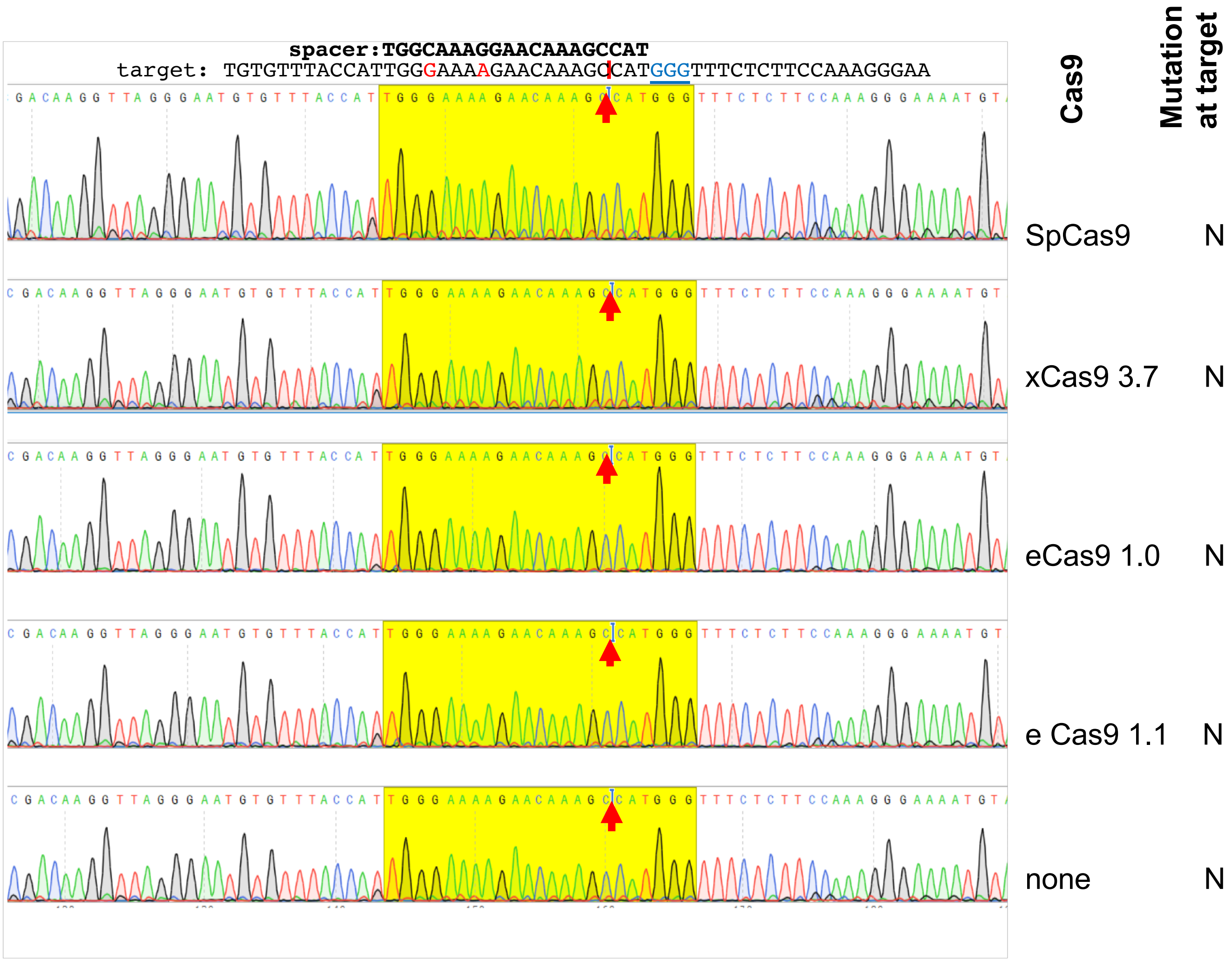
spacer:TGGCAAAGGAACAAAGCCAT
target: TGTGTTTACCATTGGGAAAAGAACAAAGCCATGGGTTTCTCTTCCAAAGGGAA
Cas9
Mutation at target
SpCas9
N
xCas9 3.7
N
eCas9 1.0
N
e Cas9 1.1
N
none
N

AT2G32730

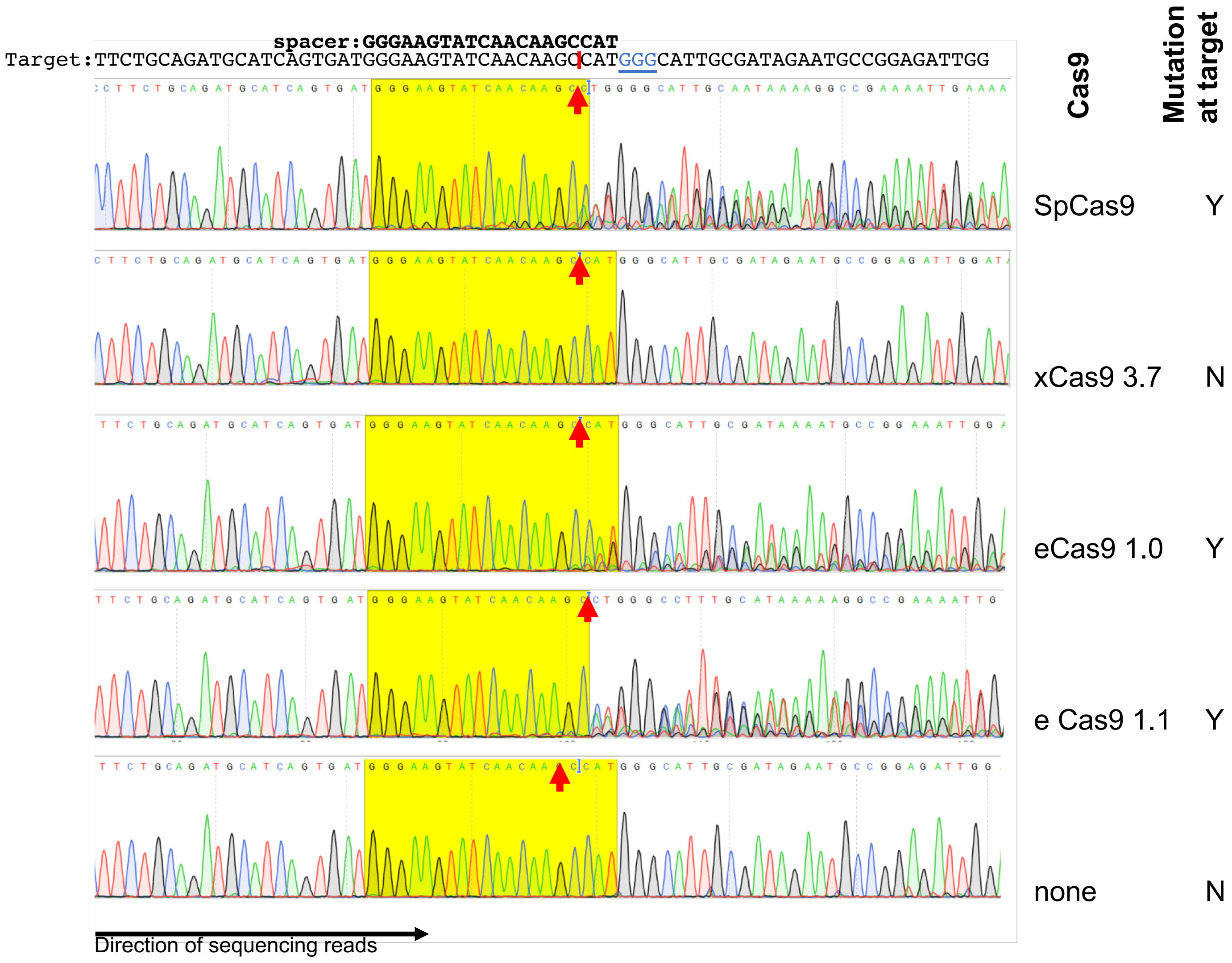
spacer:GGGAAGTATCAACAAGCCAT
Target:TTCTGCAGATGCATCAGTGATGGGAAGTATCAACAAGCCATGGGCATTGCGATAGAATGCCGGAGATTGG
Cas9
Mutation at target
SpCas9
Y
xCas9 3.7
N
eCas9 1.0
Y
e Cas9 1.1
Y
none
N
Direction of sequencing reads

AT1G04810

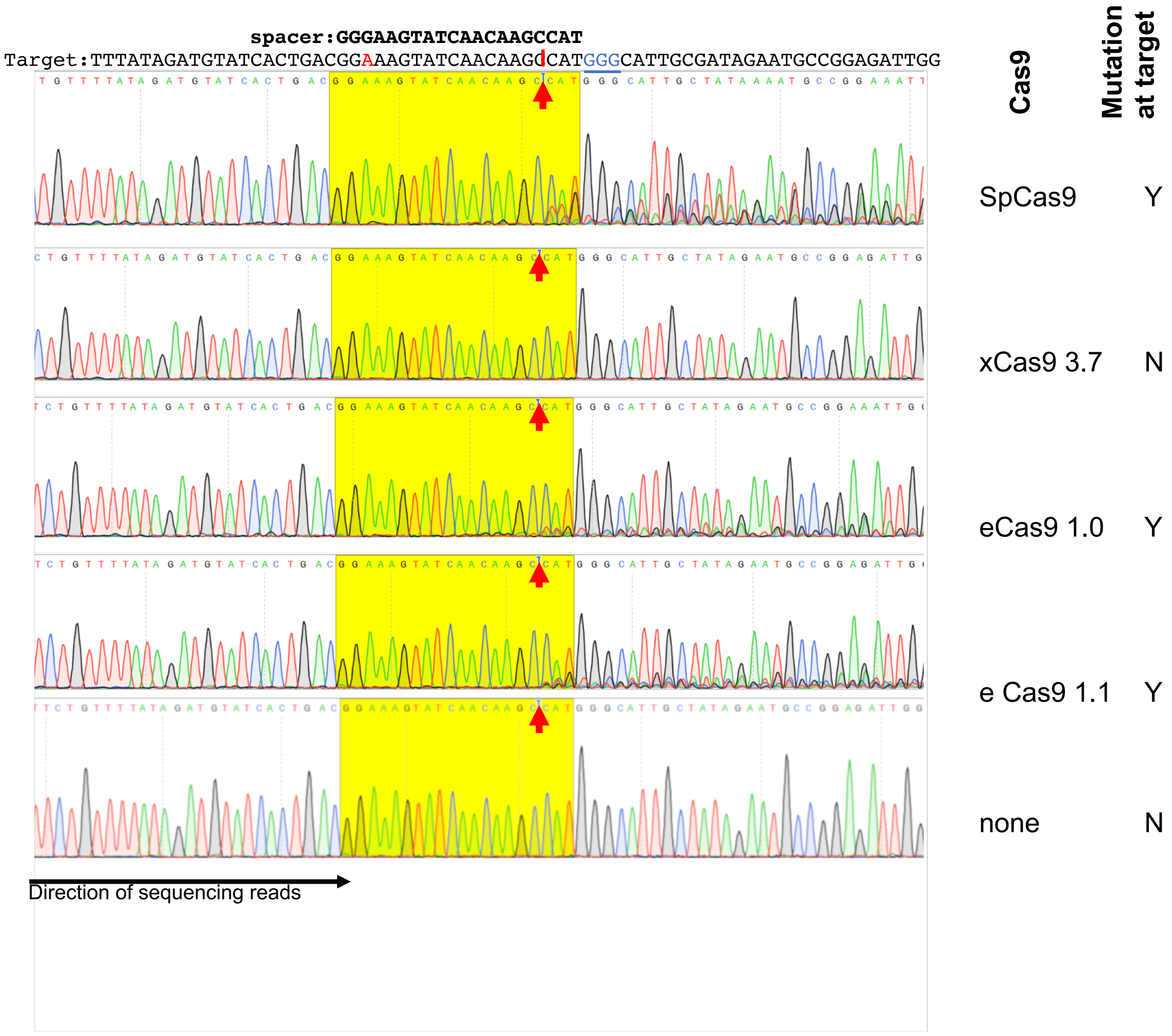
spacer:GGGAAGTATCAACAAGCCAT
Target:TTTATAGATGTATCACTGACGGAAAGTATCAACAAGCCATGGGCATTGCGATAGAATGCCGGAGATTGG
Cas9
Mutation at target
SpCas9
Y
xCas9 3.7
N
eCas9 1.0
Y
e Cas9 1.1
Y
none
N
Direction of sequencing reads

AT5G27030

spacer:GGGAAGTATCAACAAGCCAT

Target:GCAAACCTAAAAAGACGAACATCCAAGCAACAAGCCATGGGAAAAAGAATCTTTGGGAC

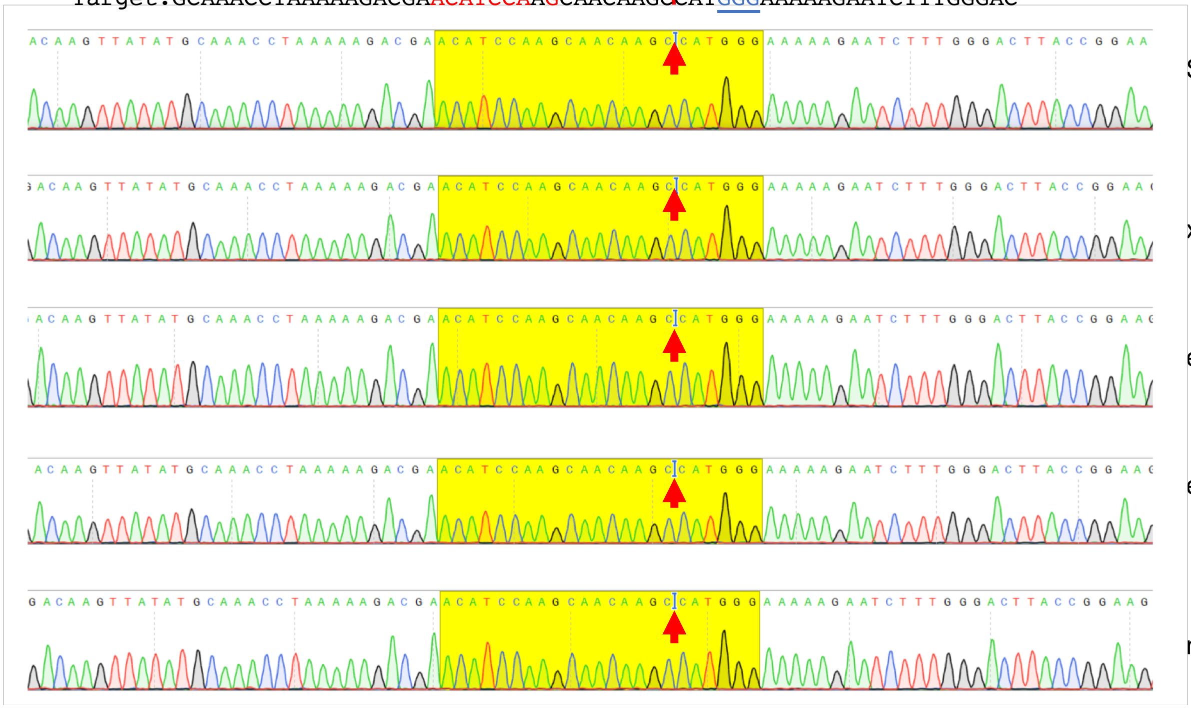

Cas9	Mutation at target
SpCas9	N
xCas9 3.7	N
eCas9 1.0	N
e Cas9 1.1	N
none	N

AT3G14720

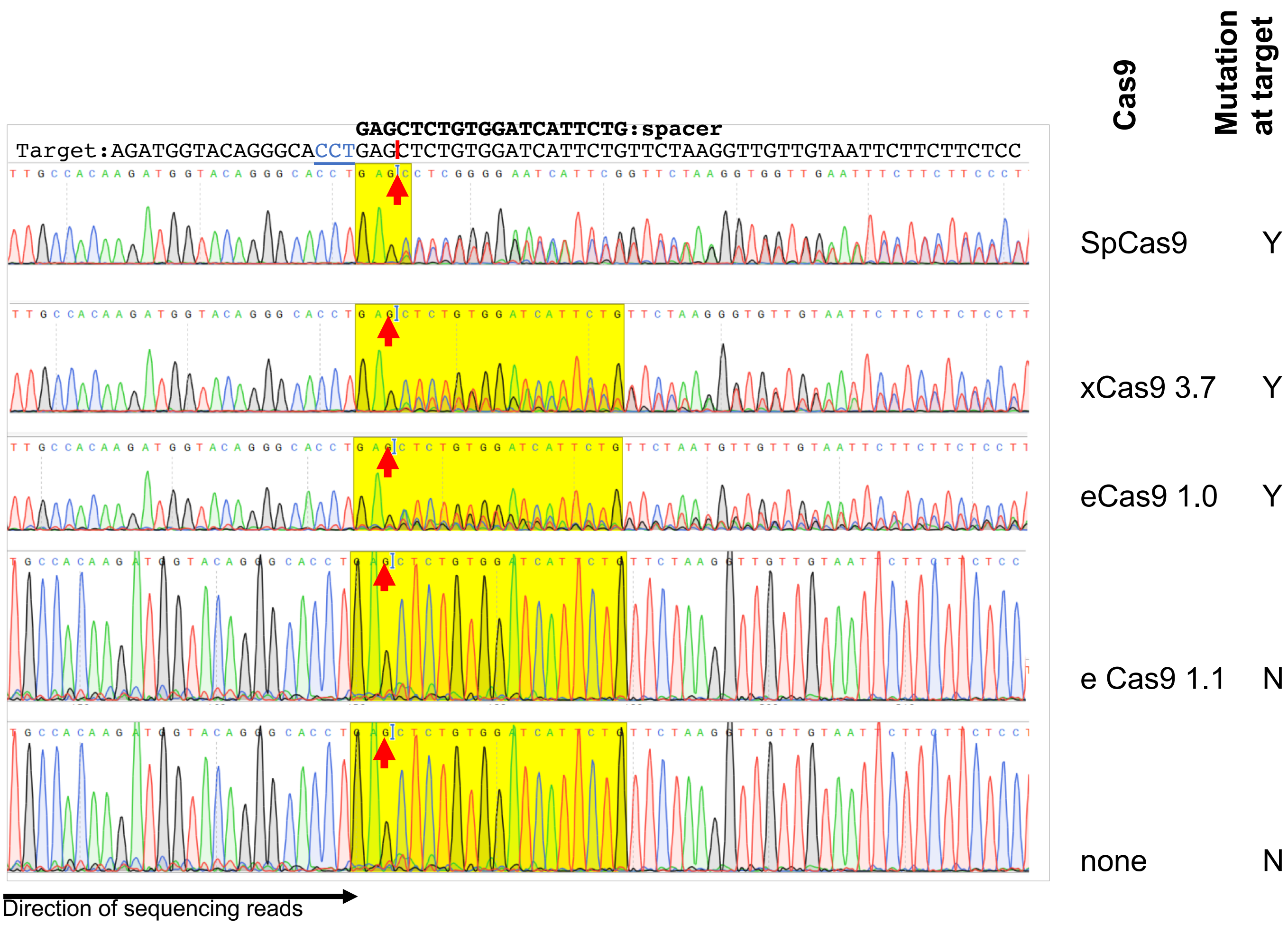
Cas9
Mutation at target
GAGCTCTGTGGATCATTCTG:spacer
Target:AGATGGTACAGGGCACCTGAGCTCTGTGGATCATTCTGTTCTAAGGTTGTTGTAATTCTTCTTCTCC
SpCas9 Y
xCas9 3.7 Y
eCas9 1.0 Y
e Cas9 1.1 N
none N
Direction of sequencing reads

AT1G53510

GAGCTCTGTGGATCATTCTG:spacer

Target:CAAGATGGTATAGAGACCTGAGCTCTGTGGATCATTCTTTTCGAAGGTAGGTGTTACTGTGGA

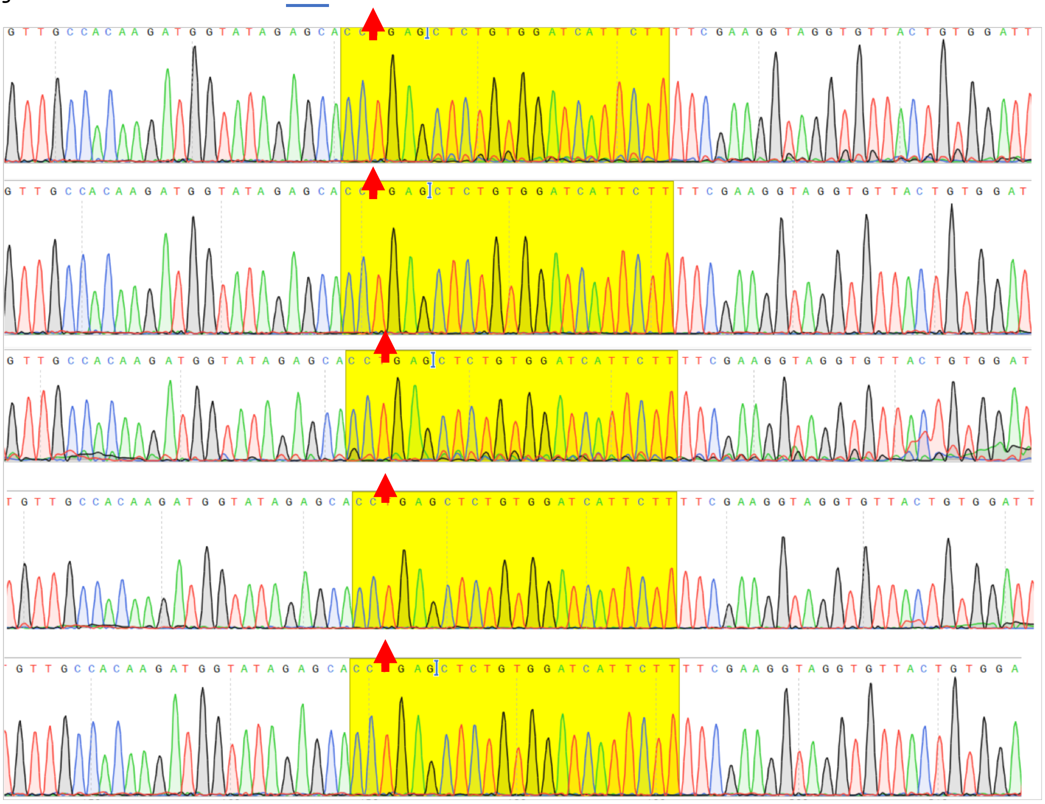

Cas9	Mutation at target
SpCas9	Y
xCas9 3.7	N
eCas9 1.0	Y
e Cas9 1.1	N
none	N

Direction of sequencing reads

AT5G16540

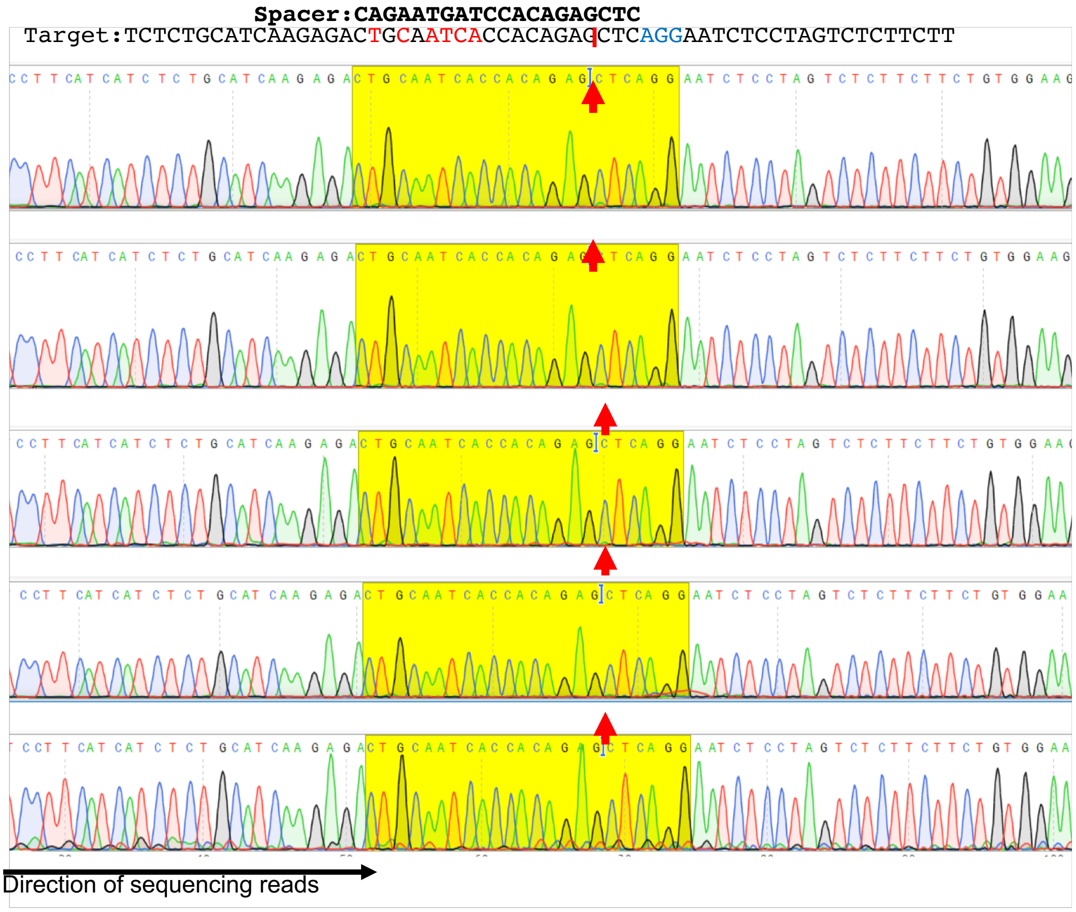

Cas9	Mutation at target
SpCas9	N
xCas9 3.7	N
eCas9 1.0	N
e Cas9 1.1	N
none	N

AT5G10620

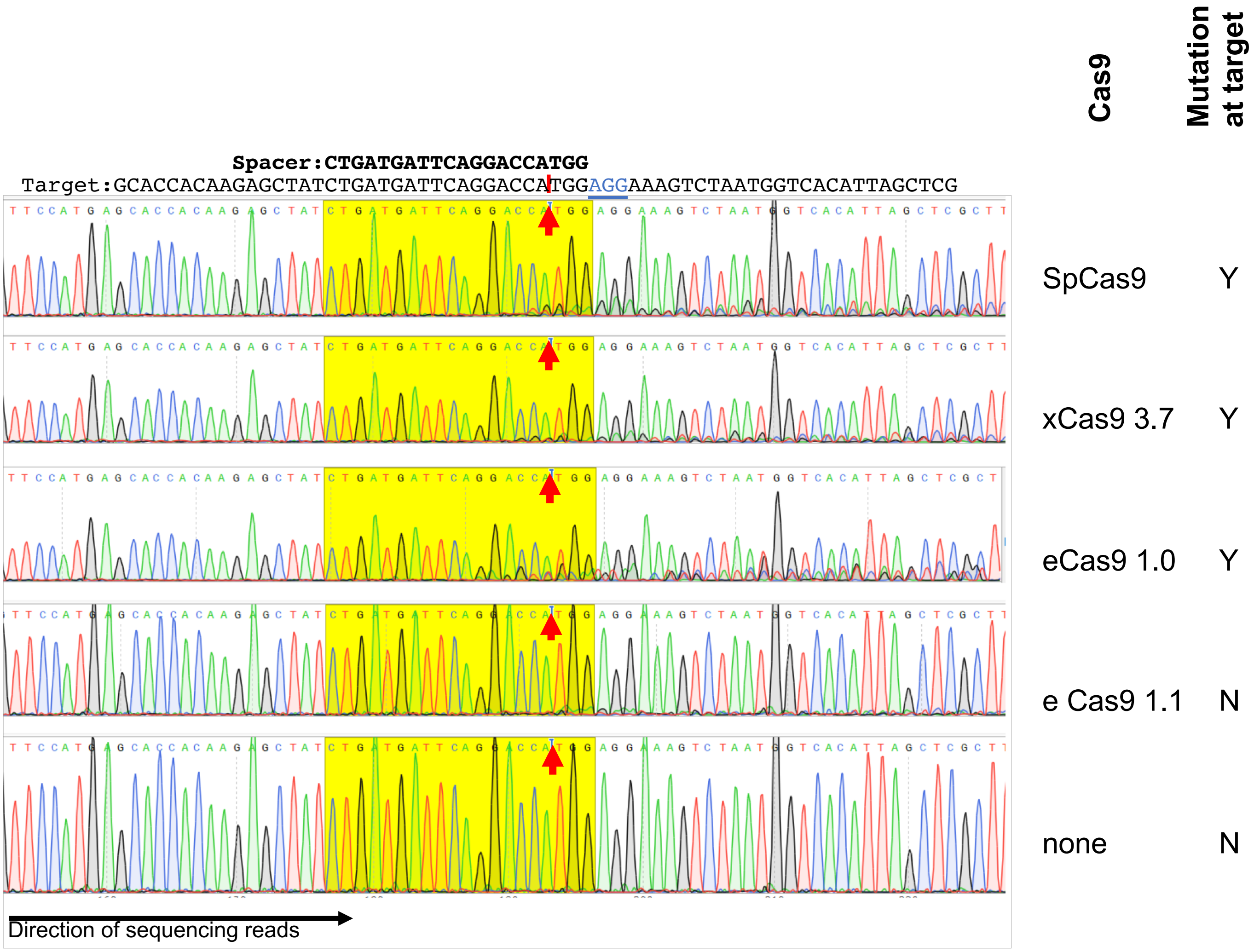
Cas9
Mutation at target
Spacer:CTGATGATTCAGGACCATGG
Target:GCACCACAAGAGCTATCTGATGATTCAGGACCATGGAGGAAAGTCTAATGGTCACATTAGCTCG
SpCas9
Y
xCas9 3.7
Y
eCas9 1.0
Y
e Cas9 1.1
N
none
N
Direction of sequencing reads

AT5G10605

Spacer:CTGATGATTCAGGACCATGG

Target:AGTACCACAAGAGCTATATGATGATTCAGGACCATGGAGGAAAGTCTAATGGTCACATTAGCTCG

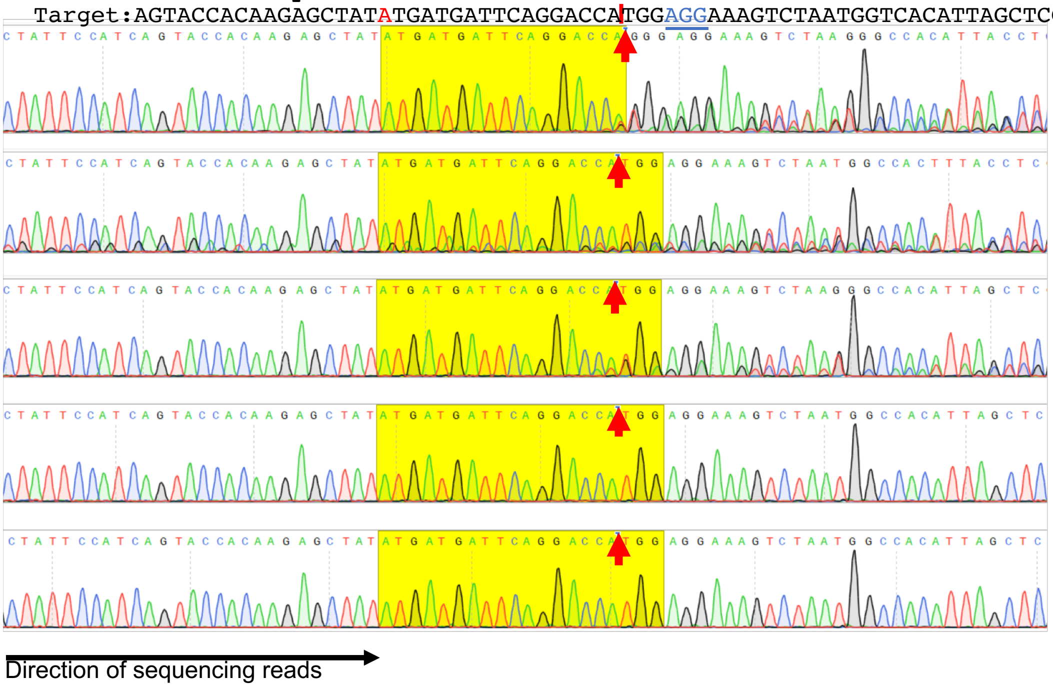

Direction of sequencing reads

Cas9	Mutation at target
SpCas9	Y
xCas9 3.7	Y
eCas9 1.0	Y
e Cas9 1.1	N
none	N

AT1G22130

Spacer:CTGATGATTCAGGACCATGG

Target:GGAGATACGAACCGGATCCAATAAGATTCACGACCATGGAGGAATACGAAGTTTCTGAGAAG

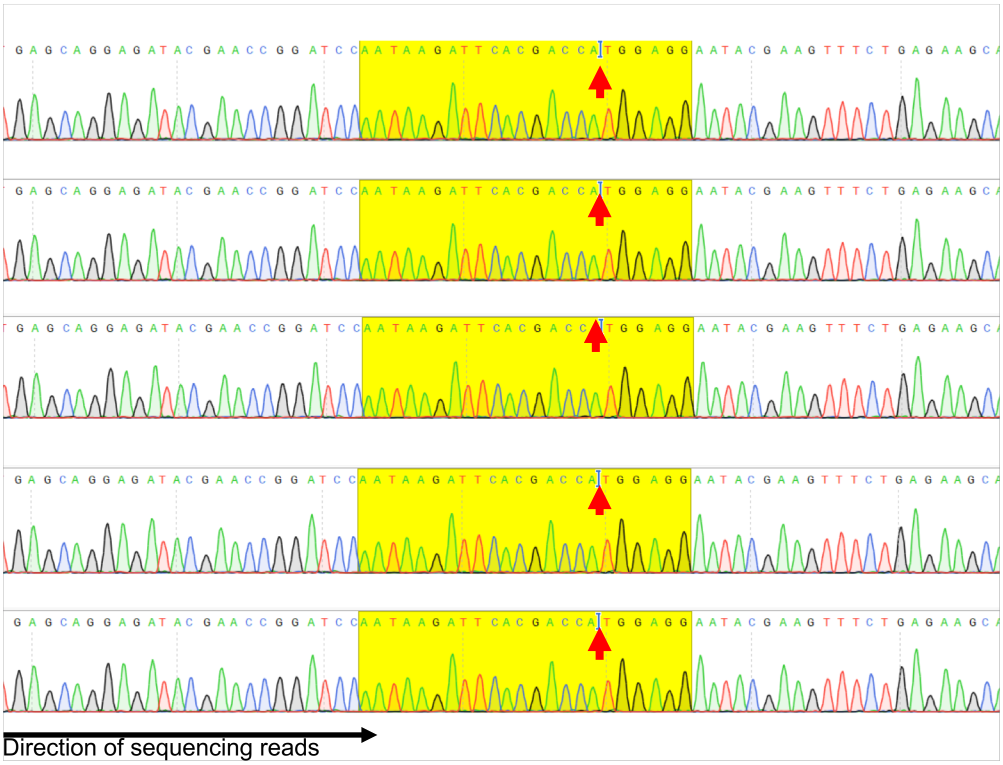

Cas9	Mutation at target
SpCas9	N
xCas9 3.7	N
eCas9 1.0	N
e Cas9 1.1	N
none	N

AT5G19360

Spacer: CCATACTTGCCATTCCATGG

Target: GACGATTGTGCAGATTATCCATACTTGCCATTCCATGGGGTTATCCACAGGGACTTCAAGCCTGAGAACT

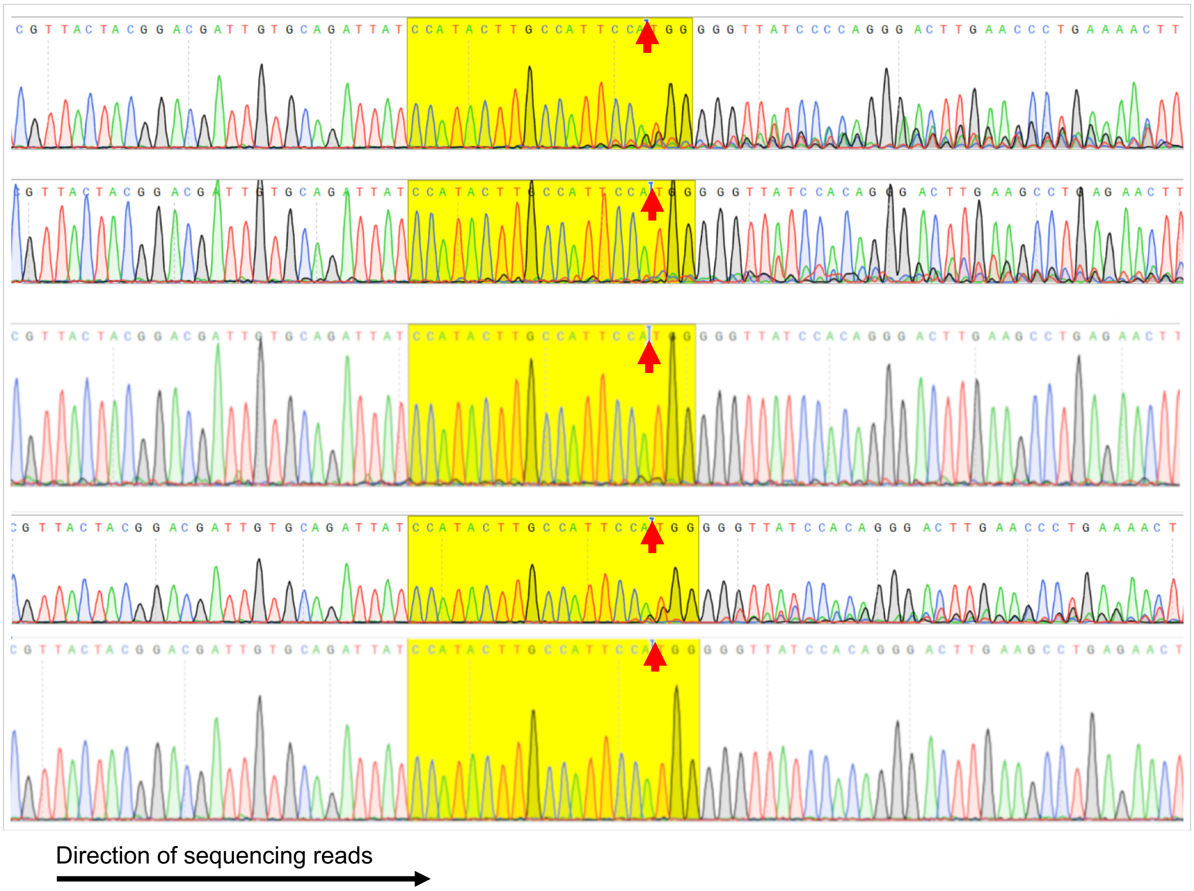

Cas9	Mutation at target
SpCas9	Y
xCas9 3.7	Y
eCas9 1.0	Y
e Cas9 1.1	Y
none	N

AT5G12180

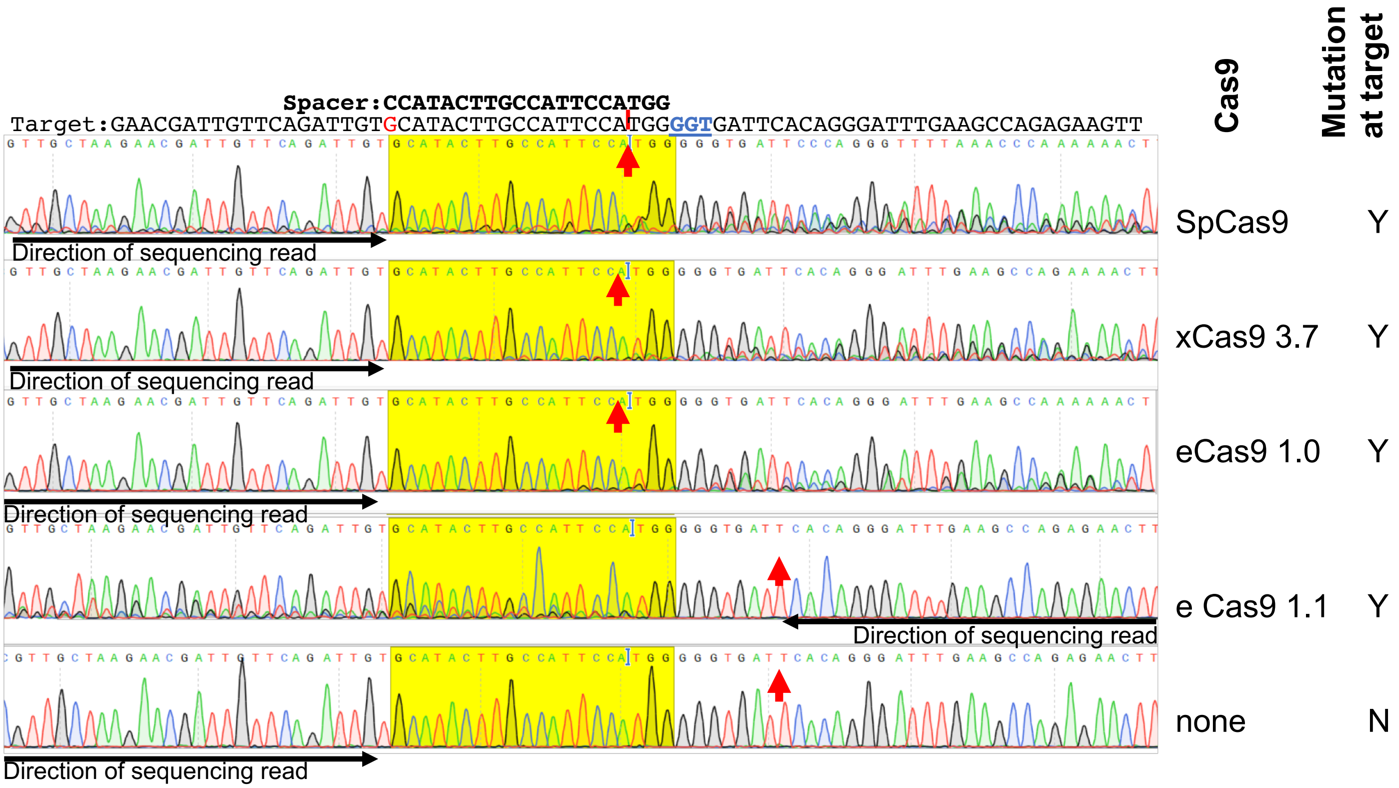
Spacer:CCATACTTGCCATTCCATGG
Target:GAACGATTGTTCAGATTGTGCATACTTGCCATTCCATGGGGTGATTCACAGGGATTTGAAGCCAGAGAAGTT
Cas9
Mutation at target
SpCas9
Y
xCas9 3.7
Y
eCas9 1.0
Y
e Cas9 1.1
Y
none
N
Direction of sequencing read
Direction of sequencing read
Direction of sequencing read
Direction of sequencing read
Direction of sequencing read

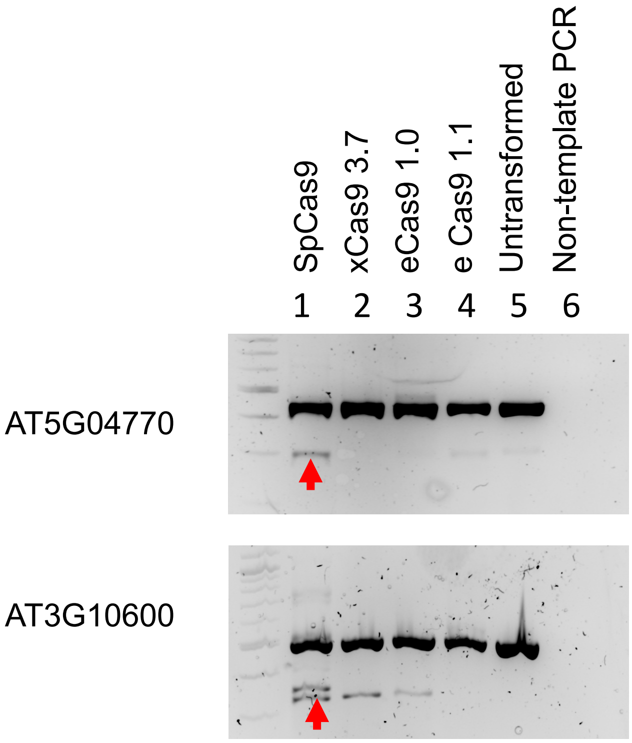


```
Spacer:     GTCGAGACGACTAGCACGGC
AT5G04770: GTCGAGACGACTAGCACGGCCGG
AT3G10600: GTAGAGACGACTAGCACGGCCGG
```

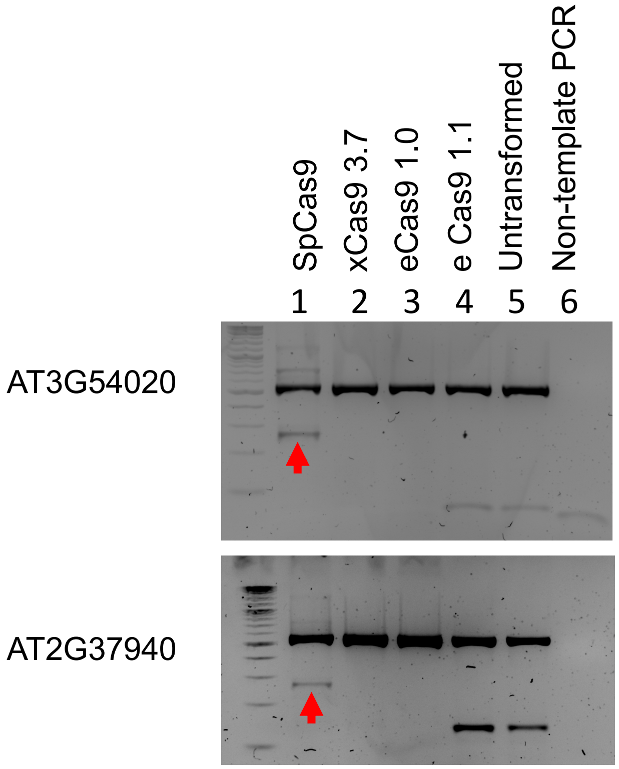


```
Spacer:     CGACAGTGATAGTTAGGGCC
AT3G54020: CGACAGTGATAGTTAGGGCCAGG
AT2G37940: CGGCAGTGATAGTTAGGGCCAGG
```

AT5G04160

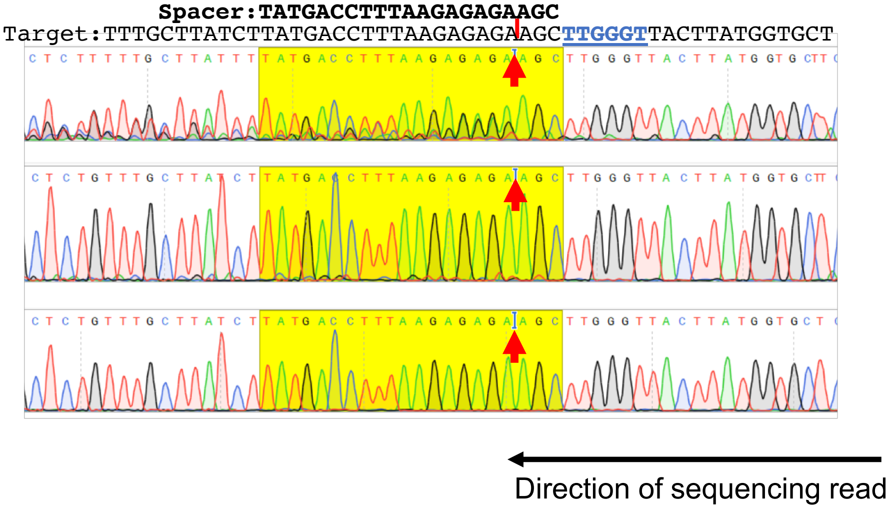

Cas9	Mutation at target
SaCas9	Y
eSaCas9	N
none	N

AT3G10290

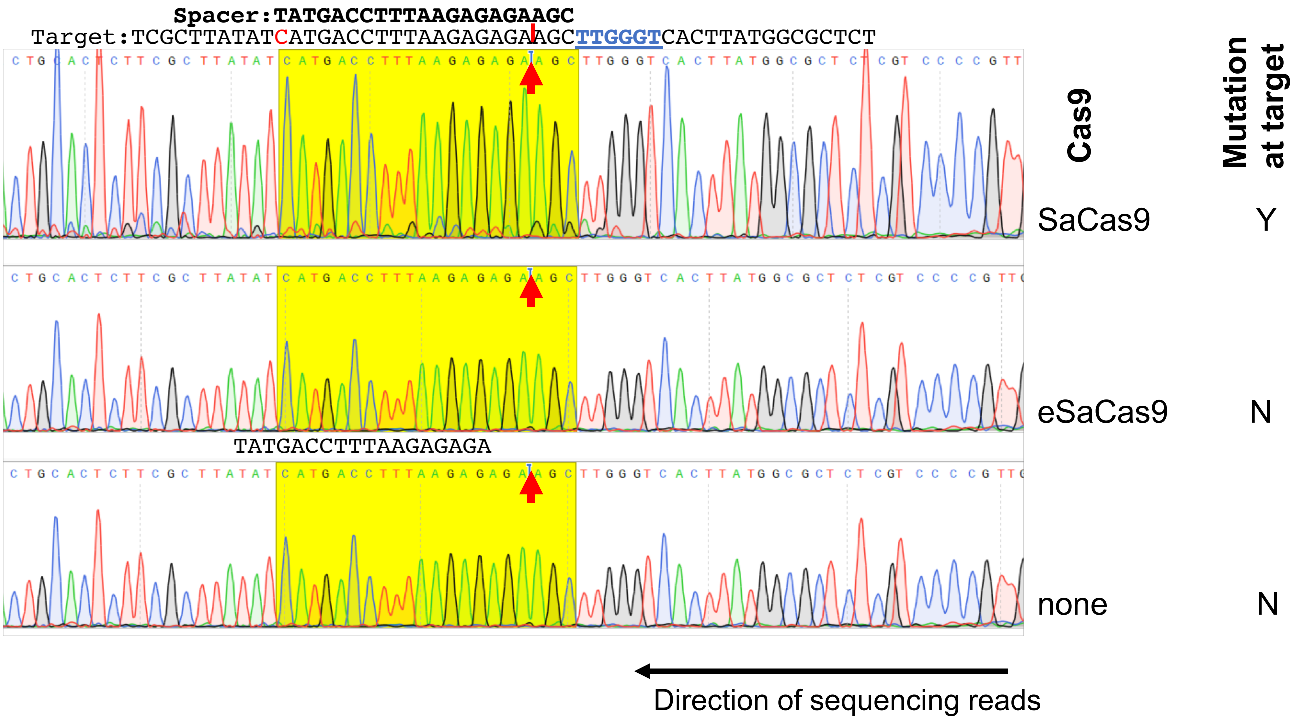
Spacer:TATGACCTTTAAGAGAGAAGC
Target:TCGCTTATATCATGACCTTTAAGAGAGAAGCTTGGGTCACTTATGGCGCTCT
TATGACCTTTAAGAGAGA
Cas9
Mutation at target
SaCas9
Y
eSaCas9
N
none
N
Direction of sequencing reads

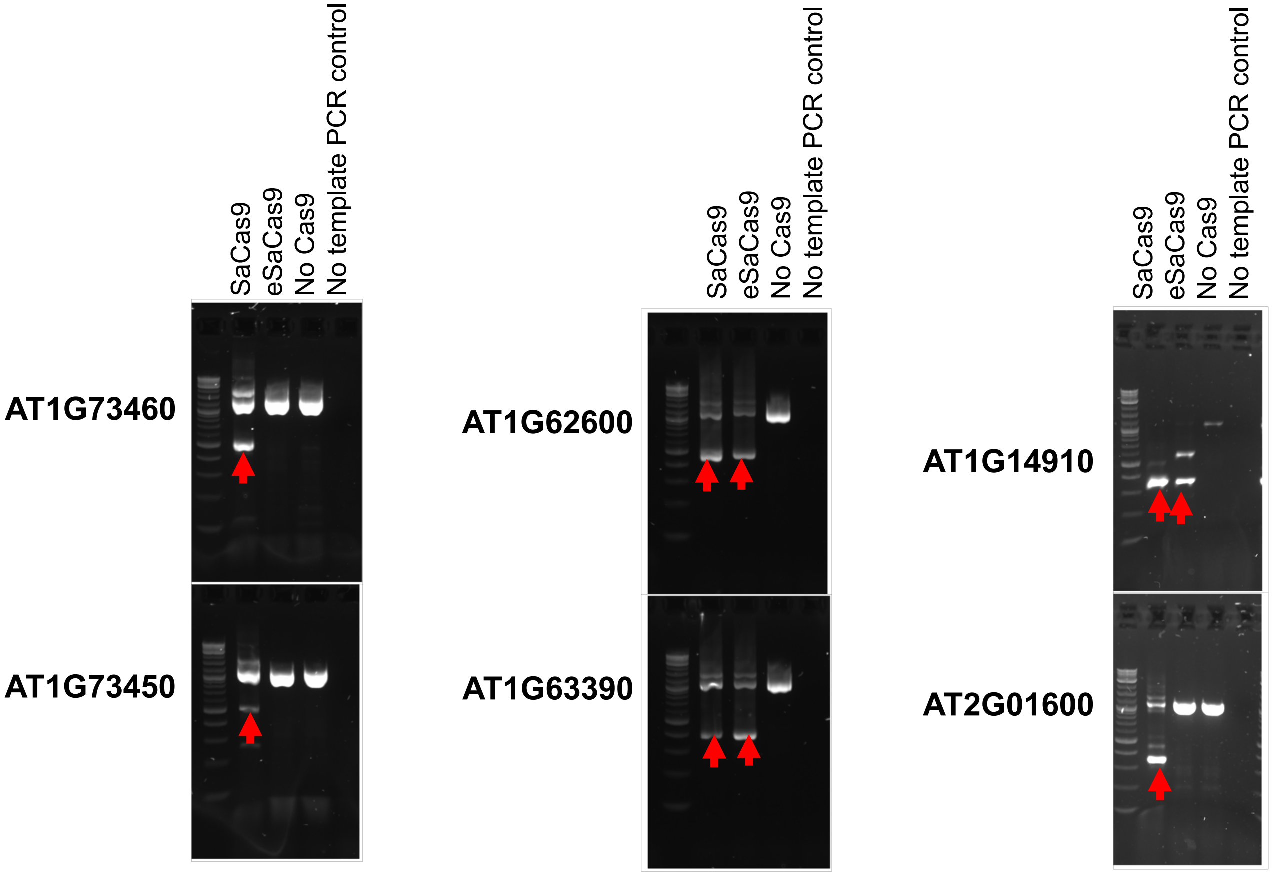

Spacer: TTCATGGTGATCAGGACTTCC
AT1G73460: TTCATGGTGATCAGGACTTCCACGGGT
AT1G73450: ATCATGGTGATCAGGACTTCCACGGGT

Spacer: GACGCTCGAATGAACAACGGA
AT1G62600: GACGCTCGAATGAACAACGGATCGGGT
AT1G63390: AACGCTCGAATGAACAACGGATCGGGT

Spacer: GCCCTTGAAATATACAAGCGT
AT1G14910: GCCCTTGAAATATACAAGCGTGCGGGT
AT2G01600: TCCCTTGAAATATACAAGCGTGCGGGT
